# Reversed functional gradient in primate prefrontal cortex: Posterior dominance and frontopolar task-related deactivation

**DOI:** 10.1126/sciadv.aea1094

**Published:** 2026-07-17

**Authors:** Kei Watanabe, Masayuki Hirata, Takafumi Suzuki

**Affiliations:** ^1^ Graduate School of Frontier Biosciences, The University of Osaka, Suita 565-0871, Japan.; ^2^ Department of Brain Physiology, Graduate School of Medicine, The University of Osaka, Suita 565-0871, Japan.; ^3^ Center for Information and Neural Networks (CiNet), National Institute of Information and Communications Technology (NICT), Suita 565-0871, Japan.; ^4^ Department of Neurological Diagnosis and Restoration, Graduate School of Medicine, The University of Osaka, Suita 565-0871, Japan.

## Abstract

The frontopolar cortex (FPC) is thought to coordinate the more posterior lateral prefrontal cortex (LPFC) during complex, nonroutine behaviors through high-level functions such as management of multiple goals, exploration, and self-generated decision-making. However, direct neurophysiological comparisons with other prefrontal regions are lacking, leaving the FPC’s putative dominance untested. Contrary to this view, our comparison of neuronal activity across the full anteroposterior LPFC in macaques during six distinct tasks probing these functions revealed a posterior-to-mid LPFC dominance, with resource-allocation signals, novelty- and reward-related learning signals, and modality-invariant decision-monitoring signals all showing a common posterior bias. In contrast, regardless of the task demands, FPC exhibited inflexible encoding of just-executed actions and consistent task-related deactivation resembling default-mode network activity. We identified a turning point in this graded posterior-to-anterior transition from task-positive to task-negative regions around the border between the anterior and middle thirds of the LPFC. These findings challenge the prevailing notion that the LPFC is anterior dominant across primate species and provide evolutionary constraints on theories of human prefrontal organization.

## Teaser

Single-neuron mapping flips prefrontal hierarchy, placing adaptive control posteriorly and frontopolar cortex as task negative.

## INTRODUCTION

While an understanding of the functional organization of the lateral prefrontal cortex (LPFC) is key to unraveling the neural basis of intelligent behavior, consensus remains elusive. A prominent view suggests an anterior-dominant functional gradient in the LPFC in which more anterior regions, including the frontopolar cortex (FPC; Brodmann area 10), play progressively abstract, integrative, and supervisory roles over posterior regions (*1*–*3*). Others argue that the apex of the prefrontal hierarchy lies in the mid-dorsolateral PFC (*4*, *5*) or that the LPFC does not follow a fixed hierarchical structure (*6*). Thus, it is critical to understand frontopolar function to elucidate how the broader LPFC orchestrates complex cognition, yet this understanding remains poorly developed. In macaques, a key model for human brain function, FPC studies are scarce, and the results of lesion (*7*–*10*) and neural recording studies (*11*–*13*) are not straightforward. Focal FPC lesions do not cause major deficits in standard cognitive tasks with predefined goals and relatively simple, established structure, involving memory, value, rules, or strategies (*7*–*9*). By contrast, deficits have been reported in less structured situations, including rapid novel learning (*7*, *8*). Consistent with this pattern, FPC neurons encode conventional task variables only weakly (*11*, *12*). Instead, the monkey FPC appears to be more prominently involved in complex, nonroutine situations, including (i) managing multiple goals by allocating cognitive resources (cognitive orchestration) (*8*), (ii) exploring novel environments during rapid novel learning (*7*, *13*), and (iii) monitoring “self-generated decisions” to integrate past experiences with current cues for guiding future behavior (*11*). These capacities are considered key facets of an overarching function: adaptive control of behavior in dynamic, integrative contexts (*14*, *15*).

Yet, because systematic neurobiological comparisons with other prefrontal regions are lacking, the contribution of the FPC to such complex, nonroutine behavior may have been overemphasized, leaving unresolved whether control signals for these functions peak in the FPC or in posterior PFC. Moreover, existing monkey FPC studies are scarce, use a narrow range of tasks, and often analyze only neurons prescreened for robust task-related responsiveness (*11*, *13*), making it premature to conclude that the FPC is genuinely critical for these processes. For instance, evidence for the monkey FPC’s role in cognitive orchestration comes from a single lesion study (where the effect size is compared only to that of the posterior cingulate cortex) (*8*), and underlying neuronal mechanisms are unexplored. In self-generated decision monitoring, the only prior FPC recording study focused exclusively on spatial tasks, overlooking object-based decision-making (*11*). These limitations warrant a systematic, full-sample comparison of neural activity across the entire anteroposterior (AP) LPFC to determine how these functions are distributed.

Here, we examined neuronal activity across the full anteroposterior LPFC while monkeys performed six tasks designed to probe the proposed functions of the FPC. These comprised (i) unimodal and multimodal dual tasks taxing cognitive orchestration (experiments 1 and 2), (ii) two rapid learning tasks engaging exploratory behavior (experiments 3 and 4), and (iii) spatial and object self-generated decision-making tasks requiring integration of internal and external information for behavioral adaptation (experiment 5). All tasks, except for the spatial self-generated decision task, are new to FPC electrophysiology; several are novel variants of classic lesion paradigms previously used to probe FPC function. Since they use both spatial and object-based information and multiple motor outputs (saccadic and manual responses), these experiments provide a rigorous test of whether the FPC plays a dominant role in the broader LPFC. We found that adaptive-control signals for all these functions peaked in the posterior-to-mid LPFC. By contrast, FPC activity was markedly inflexible, encoding predominantly the spatial location of just-executed actions regardless of task demands. Further analyses revealed an anteriorly intensified task-related deactivation extending into the FPC, suggesting the emergence of a default-mode network (DMN) (*16*, *17*) node within the macaque anterior LPFC.

## RESULTS

### Localization of neural activity

**Fig. 1.**
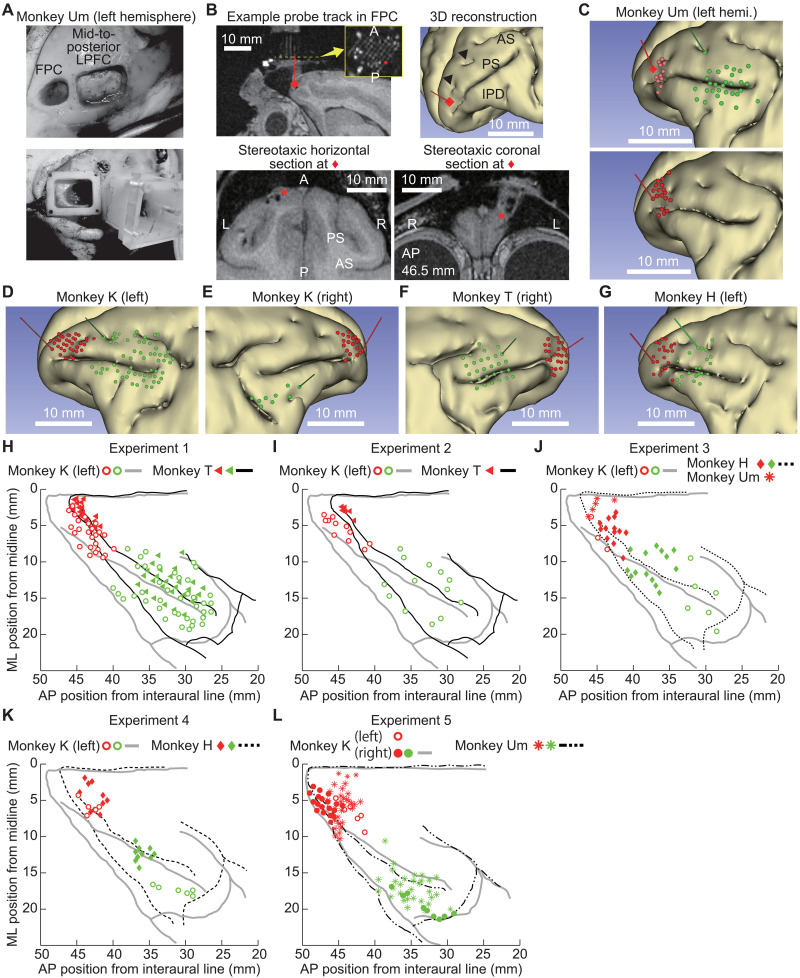
Recording sites. (**A**) Craniotomies (top) and recording chambers (bottom) over the FPC and mid-to-posterior LPFC in monkey Um. (**B**) Example probe track in FPC (monkey Um). Top left: A probe inserted through the highlighted grid hole (red) reaches the cortical surface at the red diamond; scale bar, 10 mm. Inset: 1.5× enlarged view of the grid along the yellow dotted line. Top right: The same probe trajectory (red line) and cortical entry point (red diamond) shown on a three-dimensionally reconstructed brain. PS, principal sulcus; AS, arcuate sulcus; IPD, inferior principal dimple. Black triangles: rostral, anterior supraprincipal dimple (aspd); caudal, posterior supraprincipal dimple (pspd). Bottom: Stereotaxic horizontal and coronal sections at the entry point (red diamond). A, anterior; P, posterior; L, left; R, right. (**C**) Top: Reconstructed recording sites in monkey Um. The brain is aligned to stereotaxic coordinate space and tilted 30° toward the viewer around the AP axis. Green and red diagonal lines indicate probe-insertion angles for the two chambers. Bottom: Because the FPC chamber was relocated once in this monkey, recording sites were re-reconstructed using updated MRI images. Pink and red circles denote FPC-chamber sites; green circles denote mid-to-posterior LPFC (pos-PFC) chamber sites. (**D** to **G**) Same as in (C), but for the remaining three monkeys. (**H**) Recording sites for experiment 1 on top-down views of stereotaxically positioned brains of monkeys K (gray outline) and T (black outline). Axes show AP (*x*) and ML (*y*) stereotaxic coordinates relative to the interaural midpoint (origin). Right-hemisphere sites (monkey T) are flipped to appear as left. Circles and triangles denote sites in monkeys K and T, respectively; color coding as in (D). (**I** to **L**) Same as in (H), but for experiments 2 to 5. In (J) and (L), the hemisphere outline with the fewest sites was omitted for visibility.

In each daily session, we inserted a linear multielectrode probe (U-probe) into each of the two recording chambers: one targeting the FPC and the other the mid-to-posterior LPFC (Fig. 1A). We used a grid system (Fig. 1B) for precise probe placement. To reconstruct recording locations, we acquired structural magnetic resonance imaging (MRI) images with the grid filled with a contrast agent. We then determined the stereotaxic coordinates where each probe contacted the cortex, designating these as the recording site for the neurons it recorded. For example, the probe track in Fig. 1B (red line, top left) contacted the cortex (red diamond) at stereotaxic coordinates of AP 46.5 mm and mediolateral (ML) 5.0 mm in the left FPC of monkey Um. Figure 1C shows all reconstructed recording sites within the FPC and mid-to-posterior LPFC chambers of this monkey, aggregated across experiments. Figure 1 (D to G) shows the recording sites for the remaining three monkeys (four hemispheres). Figure 1 (H to L) shows the recording sites organized by experiment. Neurons recorded from the FPC chamber are referred to as FPC neurons, and those from the mid-to-posterior LPFC chamber are pos-PFC neurons. Beyond these labels, we also examined changes in neuronal activity in 1-mm resolution along the AP axis. We recorded all neurons encountered without any preselection.

### Experiment 1: Testing the cognitive orchestration hypothesis in the monkey FPC

**Fig. 2.**
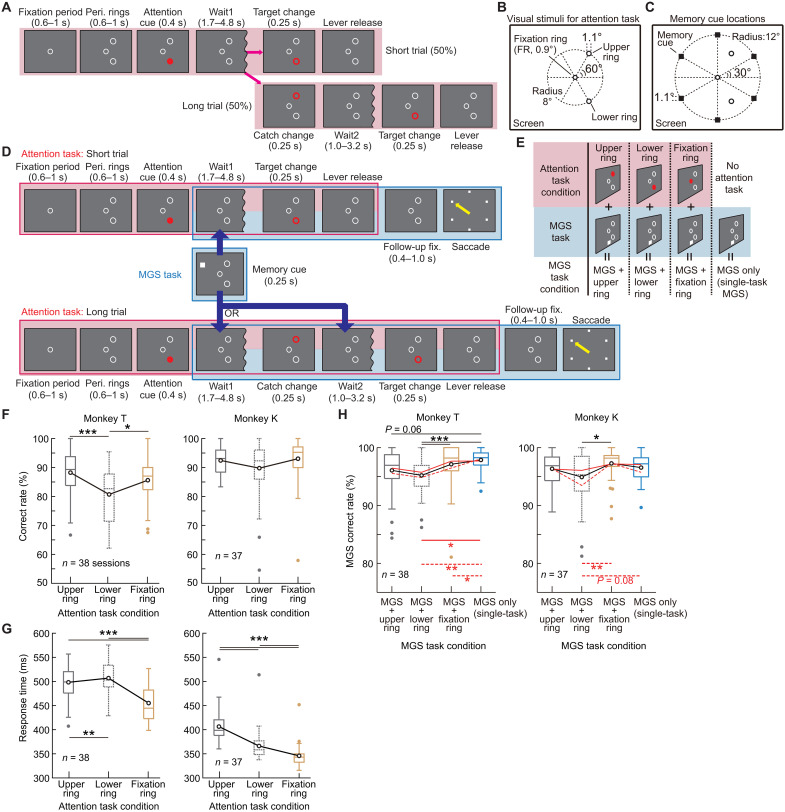
Experiment 1: Behavioral task and performance. (**A**) Event sequence of the attention task. (**B**) Location of visual stimuli in the attention task. (**C**) Location of memory cue. (**D**) Insertion of a memory cue (middle row) into either the short trial (top row) or long trial (bottom row) of the attention task. (**E**) Attention–MGS task combinations across the three dual-task MGS conditions and one single-task MGS condition. (**F**) Behavioral performance. Distribution of session-by-session percent-correct rates in the three attention task conditions for monkeys T (left) and K (right), shown as boxplots. In each box, the horizontal line and open circle represent the median and mean, respectively. The box edges mark the lower and upper quartiles; whiskers extend to the most extreme values within 1.5 interquartile ranges. Colored dots indicate outliers. Asterisks indicate *P* values. *P < 0.05, **P < 0.01, ***P < 0.001 (paired-permutation test, Holm-Bonferroni corrected). (**G**) Same as in (F), but for session-by-session mean RTs. (**H**) Distribution of session-by-session percent-correct rates in the three dual-task MGS conditions and in the single-task MGS condition for monkeys T (left) and K (right). The solid black line graph shows the mean values when all trials (regardless of memory-delay duration), whereas the solid and dashed red line graphs show means for trials with short (≤3.0 s) and long (>3.0 s) memory delays, respectively. Memory delay was defined as the interval from memory-cue offset to the go signal. Lower solid and dashed red horizontal lines indicate the results of statistical tests for short and long memory delay trials, respectively.

To examine the FPC’s contribution and to characterize the anteroposterior gradient in cognitive orchestration, we recorded the activities of 2072 single neurons (FPC, *n* = 853; pos-PFC, *n* = 1219; see table S1 for the number of neurons recorded in each monkey) in two monkeys performing a dual task (Fig. 2). The task required simultaneous performance of a visuospatial attention task and a memory-guided saccade (MGS) task (*18*, *19*). Recording sites spanned AP 26.5 to 46.1 mm (Fig. 1H).

Trials of the dual task started with the attention task, initiated by the monkey’s lever press, which triggered the onset of a central fixation ring. After a brief fixation (Fig. 2A, “fixation period”; 0.6 to 1.0 s), two peripheral rings (upper and lower rings; Fig. 2B) appeared in the contralateral visual hemifield relative to the recording hemisphere (Fig. 2A, “peripheral rings”). Fixation on fixation ring was required throughout the trial. Subsequently, an attention cue (red filled circle) appeared for 0.4 s on one of the three rings, indicating the to-be-attended target ring for that trial (the upper ring, lower ring, or fixation ring condition). After a random-length first waiting period (Wait1, 1.7 to 4.8 s), in 50% of trials (short trials), the color of the target ring briefly changed to red (target change), requiring the monkeys to release the lever within 0.8 s from the change (lever release). In the remaining 50% (long trials), the first waiting period ended with a color change in one of the two nontarget rings (catch change), to which the monkey was prohibited from responding. The monkeys then waited an additional 1.0 to 3.2 s during the second waiting period (Wait2 period) before the target change. In the single-task attention trials (i.e., trials without MGS-task insertion), a correct lever release was rewarded with a drop of juice, either immediately (50% of trials) or after an additional 0.4 to 1.0 s of central fixation (50%; randomly selected per trial).

In two-thirds (66.7%) of randomly selected attention task trials, an MGS memory cue (0.25 s; Fig. 2D) was inserted partway through the trial, during either the first or second waiting period, at one of six far-peripheral locations (Fig. 2C), thereby creating dual-task MGS trials. MGS insertion was balanced equally across short and long attention task trials, each accounting for 33.3% of all attention task trials. The memory cue appeared at a random time after attention-cue offset (1.0 to 4.1 s in short trials; 1.0 to 5.0 s in long trials). The monkeys were required to remember the cued location while continuing the attention task. After completion of the attention task (lever release) and a follow-up fixation period (0.4 to 1.0 s), all rings disappeared and six small placeholders appeared (saccade go signal). The monkeys then made a saccade (<0.5 s) to the placeholder where the memory cue had been presented and hold fixation for 0.25 or 0.6 s (blocked) before receiving a reward [the two hold-duration conditions were introduced to assess reproducibility of a prior study (*11*) regarding FPC contributions to decision monitoring; see experiment 5 below]. Thus, during the dual-task trials, the MGS component was performed concurrently with each of the three attention target-ring conditions, yielding three dual-task MGS trial types: upper-ring, lower-ring, and fixation-ring dual-task MGS trials (labeled MGS + upper ring, MGS + lower ring, and MGS + fixation ring in Fig. 2E). As a control, in separate blocks, the monkeys performed the MGS task alone (single-task MGS). These MGS-only trials followed the same timing as the dual-task trials, with the attention task events scheduled but implemented without any corresponding visual changes on the screen. By comparing neural activity between single-task and dual-task MGS throughout the LPFC, we aimed to reveal previously unknown subregional differences in neuronal contributions to dual-task cognitive orchestration. Figure S1 shows example trial sequences for single-task and dual-task MGS.

During daily recording sessions (38 for monkey T and 22 for monkey K), a dual-task MGS block was performed between two single-task MGS blocks. Monkey K also completed 15 “frequent task-switching sessions,” where single-task MGS and dual-task MGS blocks alternated more frequently (8 ± 1.2 times per session; every 40 to 50 correct trials). Unless otherwise noted, all analyses were performed using data from all sessions. An analysis of attention task performance (Fig. 2, F and G, paired-permutation test; all *P* values Holm-Bonferroni corrected) showed that in both monkeys, response time (RT) primarily reflected task difficulty: The upper- and lower-ring conditions (detecting target changes in the periphery) were significantly more difficult than the fixation-ring condition. In dual-task MGS (Fig. 2H), monkey T (left) showed a significant performance decrement relative to single-task MGS in the MGS + lower ring condition (*P* = 6.0 × 10^−4^; paired-permutation test; all *P* values Holm-Bonferroni corrected). Performance was also reduced relative to single-task MGS in the MGS + upper ring (*P* = 0.06) and MGS + fixation ring (*P* = 0.28) conditions, but these effects did not reach significance. In monkey K (right), no significant performance decrement was observed. However, when trials with a longer memory delay period (>3.0 s; dashed red line graph) were analyzed separately, performance was reduced in the MGS + lower ring condition relative to single-task MGS (*P* = 0.08). In the combined data from the two monkeys (*n* = 75), dual-task performance showed a significant decrement in the MGS + lower ring condition (*P* = 4.0 × 10^−4^) and was also reduced in the MGS + upper ring condition (*P* = 0.07), although the latter did not reach significance. These results indicate that adding the attention task to MGS increased task complexity and coordination demands relative to MGS alone, consistent with our previous reports (*18*, *19*).

**Fig. 3.**
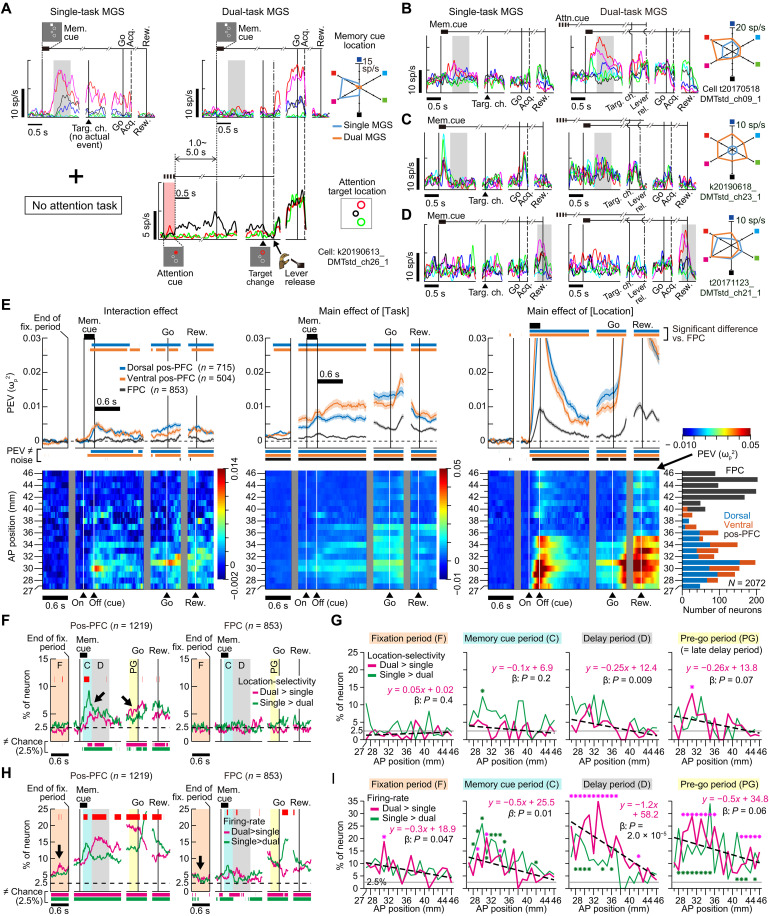
Neural activity related to cognitive orchestration across the anteroposterior LPFC. (**A**) Activity of a representative dorsal pos-PFC neuron during single-task (upper left) and dual-task MGS (upper right), aligned to memory-cue onset, target change (targ. ch.), go signal, and reward. Spike-density function colors correspond to cue locations in the polar plot, which shows mean delay-period firing rates (gray shading). Vertical dashed lines indicate mean lever-release time (lever rel.; dual-task MGS only) and mean saccadic target acquisition time (acq.). The bottom panel shows the same neuron’s activity during the attention task component of dual-task MGS. (**B** to **D**) Activity of a representative dorsal pos-PFC (B), ventral pos-PFC (C), and FPC neuron (D). (**E**) Top: Time course of population PEV for interaction (left) and main effects of task (middle) and location (right) during MGS, aligned to the end of fixation period, memory-cue onset, go signal, and reward. Shading indicates SEM. Lower horizontal bars mark time bins with significant PEV. Upper bars mark bins in which dorsal (cyan) or ventral (orange) pos-PFC differed from FPC (*P* < 0.05; FDR-corrected). Bottom: PEV time course across AP position (1-mm bins). The rightmost panel shows the number of recorded neurons per bin. Bin width = 200 ms (slid in 20 ms). (**F**) Time course of the percentages of neurons exhibiting dual>single (magenta) and single>dual (green) location-selectivity patterns. Upper red bar marks time periods in which the two percentages differed significantly. Lower horizontal bars mark periods in which each percentage differed significantly from chance (2.5%) (Fisher’s exact test, *P* < 0.05; FDR-corrected). (**G**) Changes in the percentages of dual>single (magenta) and single>dual (green) location-selectivity patterns across AP position. Regression lines/equations are shown for dual>single (magenta). Asterisks mark bins significantly above chance (2.5%) (FDR-corrected). (**H** and **I**) Same as in (F) and (G), but for dual>single (magenta) and single>dual (green) firing-rate patterns.

To examine how dual-task performance affected MGS-related activity, we conducted a two-way analysis of variance (ANOVA) with the following factors: (cue) location (six levels) and task (single-task or dual-task MGS) for each task period in each neuron. Figure 3A shows a representative dorsal pos-PFC neuron that exhibited typical location-selective activity in the delay period (gray shading, 0.4 to 1.0 s from memory cue onset) during single-task MGS (upper left), but whose selectivity was significantly reduced during dual-task MGS (upper right) (interaction, *F*_5,314_ = 15.0, *P* = 3.9 × 10^−13^). This reduction in location selectivity during dual-task MGS likely reflects this neuron’s spatial coding capacity being diverted toward target-location processing in the already ongoing attention task (Fig. 3A, bottom right), consistent with our previous observation (*18*). However, following completion of the attention task (“lever release”), in the absence of any new MGS spatial cue, this neuron regained its suppressed MGS location selectivity toward the saccadic response, even surpassing that in single-task MGS [memory reactivation; (*18*, *20*)], suggesting a reallocation of spatial coding capacity back to MGS. This activity likely reflects an adaptive distribution of cognitive resources between the two competing tasks.

The dorsal pos-PFC neuron in Fig. 3B showed an interaction effect opposite to that in Fig. 3A during the delay period (location selectivity, dual-task MGS > single-task MGS; *F*_5,344_ = 11.9, *P* = 1.3 × 10^−10^), suggesting selective participation in mnemonic processing during dual-task MGS. The ventral pos-PFC neuron in Fig. 3C exhibited a significant main effect of task during the delay period (*F*_1,241_ = 166.21, *P* = 2.8 × 10^−29^) with a higher overall firing rate across cue locations in dual-task MGS than in single-task MGS. These two dual task–enhanced activities likely contributed to dual task–specific processing. In addition, many FPC neurons (Fig. 3D) exhibited a significant effect of location only during the perireward period (gray shaded area, −0.2 to 0.3 s from reward, simple-effect *P* < 0.0002 for both single-task and dual-task MGS), consistent with prior findings in a spatial cued-strategy (CS) task (*11*).

We calculated the time course of population-averaged partial omega-squared proportion of explained variance (PEV) for each factor (Fig. 3E). A brain area that is critical for dual-task processing should show significant PEV in the interaction term and/or the factor task. However, for both, the FPC’s PEV was significantly lower than that of the dorsal and ventral pos-PFC throughout the trial (top row, two leftmost panels), indicating that the difference in neural activity between single- and dual-task conditions was significantly smaller in the FPC than in pos-PFC. A segment-by-segment analysis along the AP axis (Fig. 3E, bottom row) confirmed that the distinction was strongest at AP levels corresponding to the posterior third of the principal sulcus (AP 27 to 33 mm, spanning both intra-and extrasulcal regions), moderate in the middle third (AP 33 to 39 mm), and minimal in the anterior third and beyond (AP > 39 mm).

### Dual task–specific information processing emerges primarily in the posterior-to-mid LPFC

Dual task**–**specific information processing likely involves two neuronal response patterns. The first is a significant task × location interaction, expressed as stronger location selectivity in dual-task MGS than in single-task MGS (dual>single location selectivity; Fig. 3B). This pattern can be interpreted as content-specific recruitment, reflecting increased demand on processing task-relevant content during dual-task trials. The second pattern is a significant main effect of task, with higher overall firing rates in dual-task MGS than in single-task MGS (dual>single firing rate; Fig. 3C). This pattern reflects load-indexing recruitment, scaling with overall task difficulty or complexity, and potentially triggering dual task–specific processing in other neurons. To determine when and where these response patterns occurred, we computed, for each region, the time course of the percentages of all recorded neurons exhibiting each pattern (Fig. 3, F and H, magenta curves). For comparison, we also calculated the corresponding percentages for significant single task–preferring response patterns (single>dual location selectivity and single>dual firing rate; Fig. 3, F and H, green curves). Hereafter, we collapsed data from dorsal and ventral pos-PFC.

The results showed that in the pos-PFC (Fig. 3F, left), but not in the FPC (right), the percentage of neurons showing the dual>single location-selectivity pattern (magenta) significantly exceeded chance (2.5%) during the memory cue and delay periods (cyan and gray shaded areas) and remained above chance across much of the trial. This suggests that an add-on information-processing mechanism that operates selectively during multitasking exists only in the pos-PFC. For load-indexing recruitment (Fig. 3H), only in the pos-PFC (left) did neurons exhibiting the dual>single firing-rate pattern (magenta) significantly outnumber those exhibiting the single>dual firing-rate pattern (green) throughout the delay and pre-go (late delay) periods, indicating that only the pos-PFC showed an overall increase in firing rate in response to an increasing number of competing goals.

**Fig. 4.**
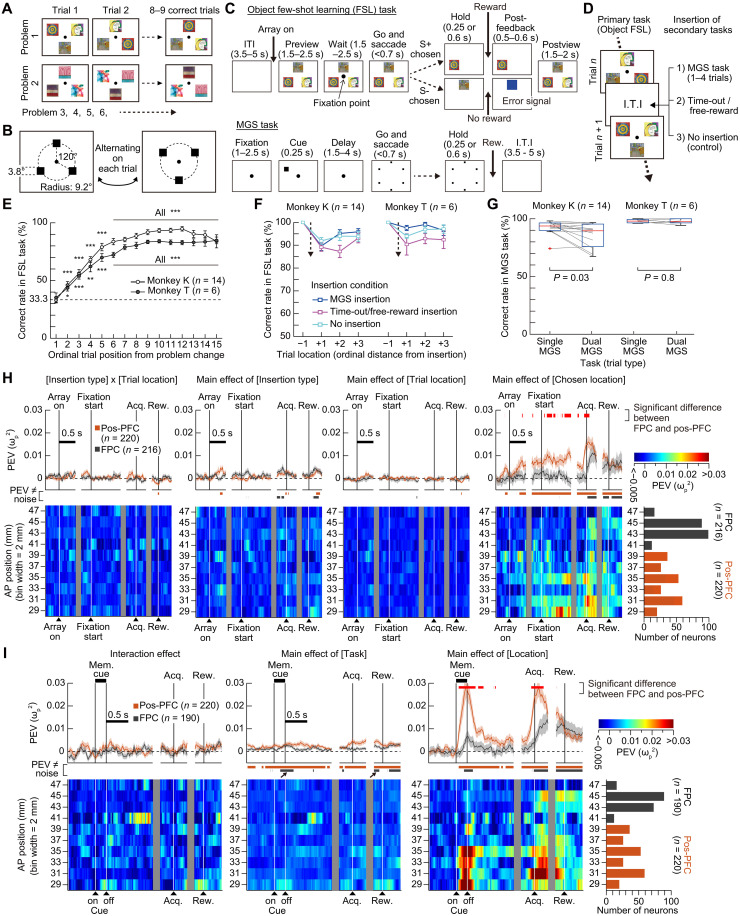
Neural activity during the serial multimodal dual task across the anteroposterior LPFC. (**A**) Progression of the object FSL task. Monkeys K and T performed an average of 36.4 and 37.7 problems per session. (**B**) Two spatial configurations for object presentation in the FSL task, alternating on each trial. (**C**) Event sequences of the FSL (upper row) and MGS (bottom row) tasks. (**D**) Insertion of a secondary task during the ITI of the FSL task. (**E**) Behavioral performance in the FSL task. (**F**) Differential effects of three secondary-task insertions on FSL performance. Dotted arrows indicate the timing of insertion. (**G**) Comparison of correct response rates between single-task and dual-task MGS. (**H**) Time course of population PEV (mean ± SEM) for the four key variables in the FSL tasks, aligned at the start of the preview period (“array on”) and wait period (fixation start), and the timing of target acquisition (acq.) and reward (rew.). Upper row: Red horizontal bars mark periods of a significant difference in PEV between the FPC (gray curve) and pos-PFC (orange curve) (*P* < 0.05, FDR-corrected). Lower colored horizontal bars mark periods when PEV was significantly different from noise in each recording area (FDR-corrected). Bottom row: Time course of PEV in each 2-mm segment along the AP axis, with the rightmost panel showing the number of recorded neurons. (**I**) Same as in (H), but for the comparison between the single-task and dual-task MGS. Note that because of a program error in the first session for monkey T, the cue location of the dual-task MGS was not recorded, and therefore, 26 FPC neurons were removed from this analysis.

Note that the opposite bias in the memory cue period (green>magenta; Fig. 3H) likely reflects global attenuation of cue-evoked visual responses during dual-task MGS, as attention is engaged by the concurrent visuospatial attention task, consistent with our previous report [figure 4a in (*18*)]. A second opposite bias (green>magenta) around the time of saccade execution (post go; Fig. 3H) likely reflects the less predictable go signal in single-task MGS due to task structure, which elicits a stronger phasic saccade-related response in single-task than dual-task MGS, again consistent with our previous report [figure 8e in (*18*)]. We therefore focused on the delay and pre-go (late delay) periods, which are temporally dissociable from external events and more directly reflect internally maintained signals. The pattern in Fig. 3H was replicated when examining raw overall firing rates (averaged across all location conditions): Population firing rates were significantly higher in the dual-task than in the single-task MGS during the delay and pre-go periods in pos-PFC (fig. S2). To our knowledge, these two types of dual task–enhanced responses constitute the first evidence of prefrontal neuronal recruitment during cognitive orchestration in macaques. They may be broadly consistent with the selective blood oxygen level–dependent–signal increase in human anterior LPFC during multitasking (*21*, *22*).

Next, for each 1-mm AP bin, we examined how the percentages of neurons exhibiting the dual>single location-selectivity pattern and dual>single firing-rate pattern varied along the AP axis during the fixation, memory cue, delay, and the pre-go periods (magenta plots in Fig. 3, G and I; analysis windows shown in Fig. 3F). During the delay and pre-go periods, the dual>single response patterns (magenta) for both location selectivity and firing rate showed an anteriorly decreasing trend (β < 0) in an ordinary least squares (OLS) regression of percentage on AP position (delay period: *P* = 0.009 for dual>single location selectivity and *P* = 2.0 × 10^−5^ for dual>single firing rate; pre-go period: *P* = 0.07 and *P* = 0.06; Fig. 3, G and I, two rightmost panels). For the dual>single firing rate pattern (Fig. 3I), significant anteriorly decreasing trend was also observed in the fixation and cue period (*P* < 0.047 for both). These results are again consistent with a limited contribution of the FPC to cognitive orchestration. Notably, only in the pos-PFC (Fig. 3H, left) did the proportion of neurons showing the dual>single firing-rate pattern (magenta) rise significantly above both chance level and the proportion showing the single>dual pattern (green), beginning in the fixation period (see arrow). This pattern suggests that, already during the preparatory period, the pos-PFC, but not the FPC, preferentially represented the higher cognitive demand expected during multitasking.

Figure 3F (left) reveals another dual task–specific phenomenon only in the pos-PFC: a dynamic shift in the relative expression of MGS-related selectivity across task phases. Consider again the representative neuron in Fig. 3A. During the cue and delay periods, it showed stronger location selectivity in single-task than dual-task MGS (neuronal dual-task interference). At the population level (Fig. 3F, left), this trend was also evident as a higher proportion of neurons exhibiting the single>dual location-selectivity pattern than the dual>single pattern during the same periods (green exceeding magenta, left diagonal arrow), indicating a transient down-weighting of MGS-related representations while the attention task was ongoing. By contrast, approaching the go time (after completion of the attention task), the same neuron (Fig. 3A) shifted to dual>single location selectivity, exhibiting memory reactivation. This shift was also evident at the population level as an increase in the proportion of neurons exhibiting the dual>single pattern near saccade go time (magenta exceeding green; right diagonal arrow in Fig. 3F, left), suggesting a relative re-emphasis of MGS-related signals. This crossover, from earlier emphasis on the attention task (green exceeding magenta) to later emphasis on MGS (magenta exceeding green), suggests a demand-dependent shift in prioritization between the two competing tasks in the pos-PFC, consistent with a putative resource-allocation process.

Together, the results of experiment 1 indicate that, contrary to the dominant view in human studies (*3*) and a previous monkey lesion study (*8*), the simultaneous performance of multiple cognitive tasks did not elicit selective activations in the monkey FPC. Instead, the orchestration process appears to be predominantly supported by the posterior-to-mid LPFC (up to approximately AP 34 mm), where significant percentages of dual task–specific activity was observed. Results were essentially unchanged when we performed the same set of analyses on neurons recorded during the aforementioned frequent task-switching sessions that controlled for potential firing-rate drift due to the block-design session structure with long blocks (fig. S3 and note S2). We obtained qualitatively identical results using normalized AP coordinates across animals (fig. S4).

### Experiment 2: Testing the cognitive orchestration hypothesis in a serial multimodal dual task

To rule out task-specific effects, we used another dual-task paradigm in which secondary tasks were inserted during the intertrial interval (ITI) of a novel-object few-shot learning (FSL) task. This structure, involving the serial presentation of tasks from different modalities, parallels that of a prior FPC lesion study (*8*). In the object FSL task, monkeys worked through a series of problems by searching for and exploiting a rewarded object (RO) stimulus (S^+^) among three objects (S^+^, S_1_^−^, and S_2_^−^; size, 3.8° on a side; Fig. 4A). The identities of S^+^, S_1_^−^, and S_2_^−^ remained the same within each problem, which lasted until the completion of eight to nine correct trials. Each problem ended with a 10- to 15-s blank interval before a new problem began, introducing three novel objects. Two different spatial configurations were used for object presentation in alternate trials, with the three objects placed randomly within each configuration (Fig. 4B).

On each trial (Fig. 4C, top), after an ITI (3.5 to 5.0 s), the monkey was presented with an object array (“array on”) and freely viewed it (“preview,” 1.5 to 2.5 s). Then, a central fixation point (FP) was presented, and the monkeys were required to fixate it. After a variable wait period (“wait,” 1.5 to 2.5 s), the disappearance of the FP prompted the monkeys to choose one of the three objects by saccade (“go and saccade”), with unchosen objects disappeared upon choice. The monkeys then fixated on the chosen object for 0.25 or 0.6 s (“hold”). Selection of S^+^ led to a reward, while selection of S_1_^−^ or S_2_^−^ resulted in an error feedback (a blue patch replacing the chosen object for 0.5 to 0.6 s; “post-feedback”). Then, the monkeys were again presented with and freely viewed the original display (“post-view”). During ITI, the monkeys were required to maintain the memory of S^+^.

On some trials, the ITI included one of three randomly assigned secondary-task conditions: MGS insertion, time-out/free-reward insertion, or no insertion (control) (Fig. 4D; Materials and Methods). In the MGS insertion condition, one to four successive MGS trials (Fig. 4C, bottom) were inserted. In the time-out/free-reward condition, a blank screen was shown for 40 to 50 s, during which one to four free rewards were delivered at random intervals, triggering attention-drawing events [as in the insertion of “reward consumption” task in the previous FPC lesion study; (*8*)]. These two insertion conditions constituted the dual-task FSL condition. In the no-insertion condition (control), nothing was inserted (i.e., single-task FSL). In addition to this comparison between single- and dual-task FSL, to complement experiment 1, we introduced a block of single-task MGS before and after the FSL block (70 to 100 trials each) and compared neural activity between these single-task MGS against the MGS trials inserted during the FSL task (dual-task MGS).

An analysis of the FSL performance showed that monkeys K and T showed significantly above chance (33%) correct rate from the second trial after problem change, indicating FSL of novel stimulus-reward (S-R) associations in each problem [Fig. 4E; one-sample *t* test, false discovery rate (FDR)–corrected]. To examine the effect of secondary-task insertion on FSL performance, we analyzed correct rate changes before and after insertion. Since our focus was how the memory of S^+^ was affected by the secondary tasks, we only analyzed cases where the trial immediately before insertion was correct. Different insertion conditions led to varying performance decrements in the postinsertion FSL trials (Fig. 4F).

In both monkeys, the time-out/free-reward insertion produced the largest performance decrement, whereas the MGS insertion produced a similar decrement to the no-insertion control. This differential effect was not significant when the monkeys were analyzed separately (two-way ANOVA interaction between insertion type and trial location: monkey K, *F*_6,78_ = 2.0, *P* = 0.08; monkey T, *F*_6,30_ = 1.5, *P* = 0.22), likely because of the limited number of sessions (*n* = 14 and 6 for monkeys K and T, respectively). In the combined dataset (*n* = 20), both the interaction and the main effect of insertion type reached significance (interaction, *F*_6,114_ = 2.2, *P* = 0.049; insertion type, *F*_2,38_ = 4.7, *P* = 0.01). Between single- and dual-task MGS (Fig. 4G), the combined dataset (*n* = 20) showed a significant dual-task performance decrement (paired *t* test, *P* = 0.02). Individual-monkey analyses yielded *P* = 0.02 for monkey K (*n* = 14) and *P* = 0.70 for monkey T (*n* = 6).

We recorded activity from 216 FPC and 220 pos-PFC neurons (Fig. 1I). To assess dual task–specific representations, we computed the time course of population-averaged PEV in the object FSL task, using a three-way ANOVA with factors: insertion type (three levels), trial location (pre- or postinsertion), and chosen location (six levels) (Fig. 4H). If the monkey FPC is involved in dual task–specific processing, we would expect a significant interaction (insertion type × trial location) and/or a simple main effect of insertion type in the postinsertion trials where the task diverged into a single or serial dual-task format. However, in both the FPC and pos-PFC, significant PEV was absent in both the above interaction term (Fig. 4H, leftmost panel) and the simple main effect (fig. S5A). The only consistently significant PEV was for chosen location (Fig. 4H, rightmost panel), a task-irrelevant spatial factor, with significant PEV in the FPC (gray) emerging only after target acquisition (acq.). These findings indicate that in this particular dual-task, both FPC and pos-PFC activity play a minimal role. These observations were further validated by the PEV computed within successive 2-mm AP segments (bottom row). Calculating the percentage of significant neurons yielded nearly identical results (fig. S5B).

Next, to compare neural activity between the single-task and dual-task MGS, we calculated the population PEV using a two-way ANOVA with factors task (single versus dual) and location (six levels) (Fig. 4I). The absence of significant interaction (leftmost panel) indicates that location selectivity did not differ between the single-task and dual-task MGS in both FPC and pos-PFC. For the factor task, the FPC showed significant PEV only during two brief epochs (140 to 440 ms from cue onset and − 200 to −80 ms from reward; arrows in middle panel), whereas the pos-PFC exhibited significant PEV throughout the trial. These results indicate that discrimination between the single- and dual-task MGS was predominantly expressed in the pos-PFC, with only limited and transient discrimination in the FPC. To identify the source of this effect, we applied the same analysis as in experiment 1 (Fig. 3H); the results are shown in fig. S5D. In the pos-PFC, neurons exhibiting the dual>single firing-rate pattern again outnumbered those exhibiting the single>dual firing-rate pattern throughout the task. This indicates that only the pos-PFC consistently showed an overall increase in firing rate as the number of simultaneous goals increased (load-indexing recruitment), regardless of task-modality combination (space-space in experiment 1 versus space-object in experiment 2) or the dual-task structure (parallel versus serial). Last, the PEV for location (Fig. 4I, rightmost panel) resembled that in experiment 1. Calculating the percentage of significant neurons yielded comparable results (fig. S5C).

Together, the results from experiments 1 and 2 indicate that, despite the vastly different task settings, adaptive activity modulation from single- to dual-task conditions is predominantly observed in the pos-PFC, whereas the FPC shows markedly weaker modulation. This suggests that monkey FPC is unlikely to play essential roles in dual task–specific processes such as task coordination (*21*, *22*) and resource allocation (*8*, *14*). In the pos-PFC, the observed neural dual-task effect was smaller in experiment 2 than in experiment 1. This is likely because, in experiment 2, the serial execution of tasks with different modalities minimized neuronal competition between the tasks and reduced the need for dual-task processing (*23*). In contrast, experiment 1 required the simultaneous execution of two spatial tasks, which likely resulted in more severe competition for processing resources (*18*) and necessitated mediation by orchestration processes.

### Experiment 3: Testing the rapid novel learning hypothesis of FPC

In a previous study, an FPC lesion moderately impaired rapid learning of novel object values in a task similar to the present novel-object FSL task (*P* = 0.048, one-tailed *t* test) (*7*). Nougaret *et al.* (*13*) reported selective modulation of FPC activity during initial novel learning. In their object-in-place task, where monkeys searched for and exploited a small rewarded target embedded in novel full-screen scenes, a full-sample analysis of all 399 recorded FPC neurons [i.e., no prescreening; supplementary figure 2 in (*13*)] found that only 6.0% (24 of 399) responded more strongly to novel than familiar scenes (novelty-preferring cells), while 1.8% (7 of 399) exhibited the opposite pattern (familiarity-preferring cells). Both studies suggested that the FPC plays a leading role in the initial stage of novel learning.

**Fig. 5.**
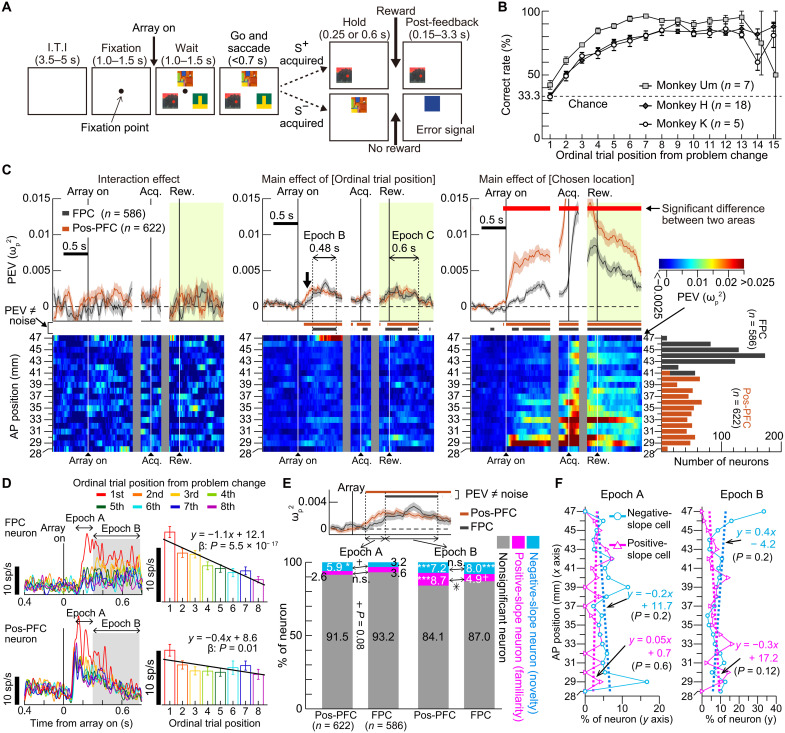
Neural activity related to novel learning across the anteroposterior LPFC. (**A**) Event sequence of the modified object FSL task. (**B**) Behavioral performance. (**C**) Population PEV time course (mean ± SEM) in FPC (gray) and pos-PFC (orange), aligned to array onset (start of the wait period), target acquisition and reward. Conventions as in Fig. 4H. (**D**) Activity of representative negative-slope neurons in FPC (top) and pos-PFC (bottom) across trials 1 to 8 after problem change (correct and error trials combined; fixation-break errors excluded). Right bar graphs show mean firing rate in epoch B (gray shading). (**E**) Percentage of negative- (cyan) and positive-slope (magenta) neurons in pos-PFC and FPC during epoch A (left) and epoch B (right). Inset: Enlarged view of the region around array onset in (C). (**F**) Change in the percentage of negative- (cyan) and positive-slope (magenta) neurons across AP position (1-mm bins). For clarity, AP position (*x* value) is plotted on the vertical axis and percentage on the horizontal axis.

However, to comprehensively assess FPC’s contribution, it is essential to compare it with other regions involved in novel learning, including the posterior LPFC (*24*–*26*). Thus, we continued recording (Fig. 1J) in the object FSL task with slight modifications (Fig. 5A, modified object FSL task): (i) omitting the preview and postview periods and secondary-task insertions and (ii) requiring monkeys to maintain central fixation from 1.0 to 1.5 s before array onset until the go signal. Three monkeys (K, H, and Um) completed an average of 47.0, 42.1, and 32.7 problems per session (*n* = 5, 18, and 7 sessions). In separate blocks, we recorded neural activity during the MGS task using the same parameters as in experiment 2, occasionally delivering free rewards during the ITI.

In the modified object FSL task, all monkeys exhibited significantly above-chance (33%) performance from the second trial after problem change, indicating rapid novel learning (Fig. 5B; one-sample *t* test, FDR-corrected). Combining data from experiments 2 and 3, we analyzed 586 FPC and 622 pos-PFC neurons. For each region, we calculated the time course of population PEV using a two-way ANOVA with factors: ordinal trial position from problem change (eight levels: first to seventh, and at least trials eighth and later combined) and chosen location (six levels) (Fig. 5C). Except for the perireward period (green shaded areas, top), both correct and error trials were analyzed (fixation-break error trials excluded). For the perireward period, only correct trials were analyzed, with the factor ordinal trial position representing the number of correct trials from problem change (first through eighth). Brain regions crucial for rapid novel learning should exhibit differential activations as a function of the ordinal trial position from problem change (i.e., significant PEV for the interaction and/or the factor ordinal trial position).

In both FPC and pos-PFC, the interaction remained nonsignificant throughout the trial (Fig. 5C, left, permutation test, FDR-corrected *P* > 0.05), indicating that chosen location selectivity did not change with novel S-R learning progression. However, for the factor ordinal trial position, two relatively long intervals showed significant PEV in both regions (middle: “epoch B,” 300 to 780 ms from array onset; “epoch C,” 0 to 600 ms from reward). This indicates that overall firing rates (averaged across all chosen locations and objects) were significantly modulated as learning progressed, suggesting that both regions reflected a transition from exploration to exploitation phases.

In epoch B, to illustrate these population-level effects at the single-neuron level, Fig. 5D shows the activity of representative FPC (top) and pos-PFC neurons (bottom) in the modified object FSL task (Fig. 5A). On the first trial of each problem, both neurons exhibited the strongest response at array onset, followed by a significant reduction in subsequent trials. Both neurons showed significantly negative slope in an OLS regression (right) with ordinal trial position as the explanatory variable and trial-by-trial firing rates in epoch B as the response variable, which was the sole criterion for classifying a neuron as a “negative-slope neuron.” Because novel objects were introduced in each problem, these activities represent object-invariant signals of novel behavioral contexts. We also identified neurons with opposite preference, those with significantly positive slopes in the OLS regression (positive-slope neuron; fig. S6, A and B), which signaled object-invariant occurrences of familiar contexts.

### Posterior-to-mid LPFC detects novel contexts earlier than FPC

One notable trend before epoch B (Fig. 5C, middle) is that, following array onset, a significant PEV increase in the pos-PFC preceded that in the FPC by 180 ms (arrow, “epoch A”; see Fig. 5E for an enlarged view). This delayed elevation suggests that the FPC may play only a subordinate role in detecting novel contexts compared with the pos-PFC, a view not tested in previous studies (*7*, *13*). To address this, we examined whether, for each epoch, the percentage of negative- and positive-slope neurons in each area exceeded chance (2.5%) and differed between areas (Fig. 5E; Fisher’s exact test, *P* values FDR-corrected for six comparisons per epoch). In epoch A (two left bars), only the percentage of negative-slope neurons in the pos-PFC (cyan) was significantly above chance (5.9%, *P* = 0.03). The other three values were nonsignificant (positive-slope neuron in pos-PFC, 2.6%; negative- and positive-slope neuron in FPC, 3.2 and 3.6%, respectively; *P* > 0.6 for all). Between areas, the percentages of negative-slope neurons in the pos-PFC (5.9%) was higher in the pos-PFC than in the FPC (3.2%), but this difference did not reach significance (*P* = 0.08, corrected), whereas the percentage of positive-slope neurons did not differ between areas (*P* = 0.6). Thus, immediately after trial onset (epoch A), only the pos-PFC, but not FPC, showed a proportion of negative-slope neurons significantly above chance.

In epoch B (Fig. 5E, two rightmost bars), all percentages were significantly above chance except for positive-slope neurons in the FPC (4.9%, *P* = 0.054); the remaining percentages were 7.2 and 8.7% in the pos-PFC (negative- and positive-slope neurons) and 8.0% in the FPC (negative-slope neurons) (*P* < 0.001 versus chance for all three comparisons). Between the areas, pos-PFC had a significantly higher percentage of positive-slope neurons than FPC (*P* = 0.02), but no such difference was observed for negative-slope neurons (*P* = 0.67). This indicates that the FPC’s contribution to novel learning catches up to the pos-PFC only at epoch B. Notably, the observed percentage of negative-slope neurons in epoch B in the FPC (8.0%, 47 of 586 neurons; rightmost bar) was similar to that of the novelty-preferring FPC cells in the study of Nougaret *et al.* (*13*) (6.0%; 24 of 399) during a comparable task period in their object-in-place task (200 to 600 ms from scene onset); the difference was not significant (*P* = 0.26). In the present study, by directly comparing pos-PFC and FPC activity in the same animals and task, we showed that in novel few-shot learning, the FPC follows, rather than precedes, pos-PFC activation. Repeating the analysis for each 1-mm AP location (Fig. 5F) revealed no evidence that the percentages of negative- or positive-slope neurons in any FPC segments exceeded chance (2.5%) during either epoch A or B (*P* > 0.1 in all 20 comparisons at each AP location, Fisher’s exact test, FDR-corrected). The seemingly high value (33%) of negative-slope neurons at AP 47-mm bin in epoch B is likely an outlier because of the small sample size (*n* = 9) (versus chance, *P* = 0.59).

One concern is that the present analysis included the data from both fixation (experiment 3) and nonfixation (experiment 2) conditions before and after array onset, potentially confounding neural activity with eye movements near array onset. This issue also applies to the prior FPC study (*13*), where fixation was not required at all during the object-in-place task. To address this, we repeated the analyses using only the experiment 3 data. The results were essentially unchanged (fig. S7 and note S3).

### Posterior-to-mid LPFC shows stronger signals related to reward prediction errors than FPC

**Fig. 6.**
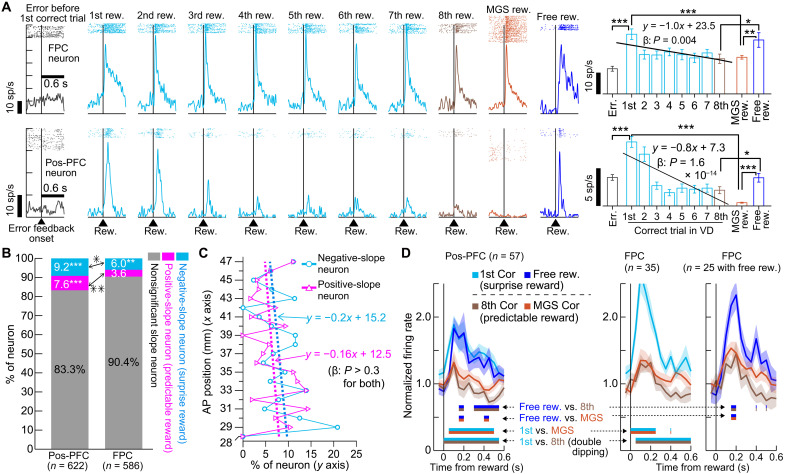
Comparison of learning-related reward signals across the anteroposterior LPFC. (**A**) Epoch C (post-reward period). Activity of representative negative-slope neuron in FPC (top) and pos-PFC (bottom) during the first to eighth correct trials after problem change in the FSL task. Responses during error trials, MGS reward, and free reward are also shown. The rightmost panel summarizes mean firing rate for each trial type in epoch C with OLS regression results. Err., error. (**B**) Same as Fig. 5E, but for epoch C. (**C**) Same as Fig. 5F, but for epoch C. (**D**) Normalized population activity of negative-slope neurons for surprise rewards (first correct trial and free reward; cold colors) versus predictable rewards (eighth and MGS correct trials; warm colors) in pos-PFC (left) and the FPC (right). Bin width, 50 ms. Shading indicates within-subject SEM (Materials and Methods). Lower bars mark time bins with significant difference between the specified conditions. For the FPC, because of a timestamp error in the first two sessions of monkey T, free-reward responses were missing for 10 negative-slope neurons; therefore, comparisons involving free reward (free versus eighth; free versus MGS; rightmost panel) were performed using the remaining 25 neurons.

In epoch C (0 to 600 ms after reward; Fig. 5C, middle), we newly identified a reward-related type of negative-slope neuron in both FPC and pos-PFC (representative neurons; Fig. 6A). Their activity decreased significantly from the first to eighth reward after problem change, with a significantly negative slope in OLS regression (rightmost panels). In addition, in both neurons, (i) delivery of free (surprise) reward elicited significantly greater activation than the eighth (predictable) reward (rightmost panels; paired permutation test, *P* < 0.03 for both neurons, FDR-corrected for four comparisons per neuron); and (ii) responses to both the first and free rewards (both surprise) were significantly greater than those to the MGS (predictable) reward (*P* < 0.004). These negative-slope (reward) neurons signal the occurrence of unexpected rewards and exhibit characteristics of a reward prediction error (RPE)–like signal that promotes learning by triggering updating of prior S-R relations (*27*). We also observed positive-slope neurons (fig. S6C), which likely signal the delivery of predictable rewards. A brain area more involved in novel learning should show a higher proportion of negative-slope (RPE-like) neurons. The pos-PFC contained a significantly higher percentage of both negative- and positive-slope neurons (9.2 and 7.6%, respectively; Fig. 6B) than the FPC (6.0 and 3.6%) (Fisher’s exact test, *P* = 0.047 and *P* = 0.005, FDR-corrected as in Fig. 5E). Across each 1-mm segment of the recording area, both neuron types showed an anteriorly decreasing trend, though not significant (Fig. 6C, *P* > 0.3 for both). Last, we compared normalized population-averaged firing rate of all negative-slope neurons in response to surprise rewards (first and free, cold colors) versus predictable rewards (eighth and MGS, warm colors; Fig. 6D). The neural responses to both types of surprise rewards were highly similar to each other and significantly exceeded those to predictable rewards in both the pos-PFC and FPC (paired-permutation test, FDR-corrected), suggesting that population activity of negative-slope (reward) neurons in both areas represents putative RPE signal.

Together, these findings indicate that, while FPC activities at array onset and after reward did distinguish exploration from exploitation during novel learning, this distinction is significantly weaker than in the pos-PFC: (i) It emerges later (array-onset response in epoch B) and (ii) involves fewer neurons (reward response in epoch C), suggesting a subordinate role for the FPC in novel learning. Notably, even in pos-PFC, negative-slope neurons comprised fewer than 10% of recorded neurons at both the array-onset and post-reward periods. Given that the ventral corticostriatal circuit, including OFC, primarily drives value-based object learning (*28*, *29*), the pos-PFC itself may have only a modest role. We also note that the present experiments were conducted after the animals were well trained on the task structure and therefore do not address the acquisition of novel task rules per se; determining the relative contributions of the FPC versus posterior PFC to novel task learning will require future studies examining the acquisition phase across the anteroposterior PFC.

### Experiment 4: Testing the rapid learning hypothesis in a small-set FSL task

**Fig. 7.**
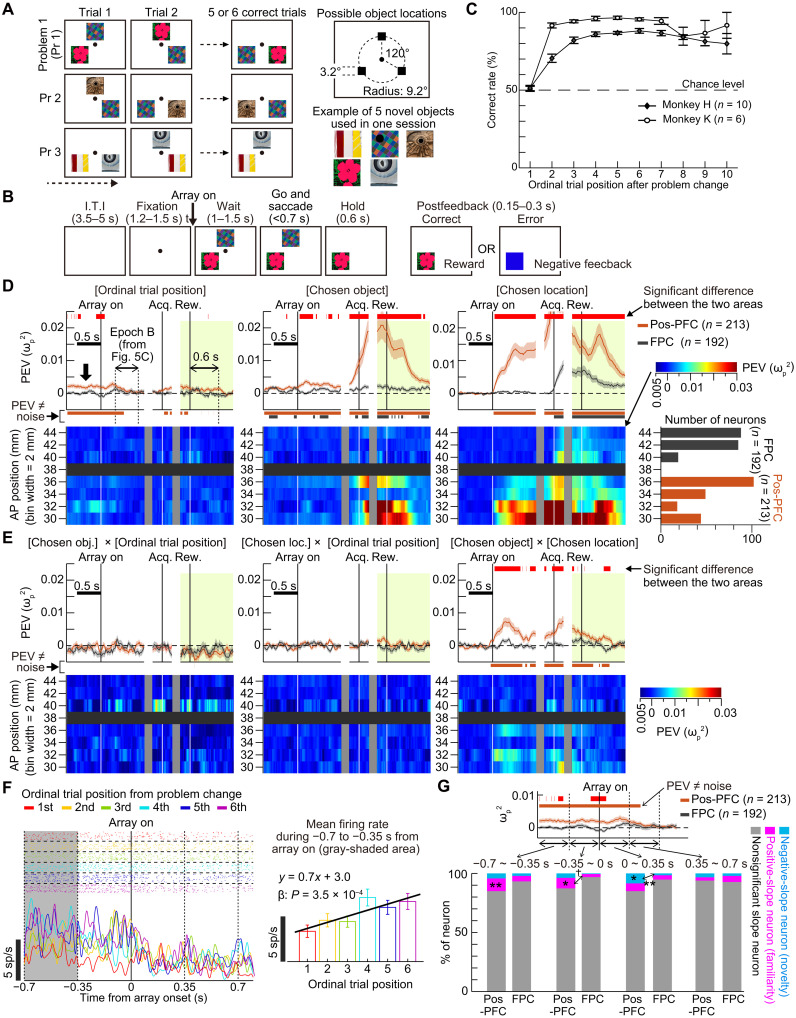
Neural activity related to learning across the anteroposterior LPFC. (**A**) Left: Task structure. Top right: Three possible object presentation locations. Bottom right: An example set of five novel objects used in one session. (**B**) Event sequence of the small-set FSL task. (**C**) Behavioral performance. Monkeys K and H completed an average of 78.7 and 69.6 problems per session. (**D**) Time course of population PEV (mean ± SEM) for the three main effects in the pos-PFC (orange) and FPC (gray). For reference, the leftmost panel shows the duration of epoch B as defined in experiment 3 (Fig. 5C, middle). Conventions as Fig. 5C. Bottom panels show PEV time courses at each 2-mm AP location. (**E**) Same as in (D), but for the first-order interaction terms. Obj., object; loc., location. (**F**) Activity of a representative positive-slope neuron in the pos-PFC across trials 1 to 8 after problem change. The right panel plots the mean firing rate during the epoch from −0.7 to −0.35 s relative to array onset (gray-shaded area in left). (**G**) Percentage of negative-slope (cyan) and positive-slope neurons (magenta) in the pos-PFC and FPC across four analysis epochs: two before and two after array onset. Inset: An enlarged view of the PEV plot for the factor ordinal trial position shown in (D) (leftmost panel) near array onset.

In experiment 3, the introduction of novel objects in each problem prevented the use of chosen-object identity as an experimental factor, so we could not directly test whether FPC neurons encode valuable objects from the earliest timing in novel learning. To address this, we further modified the object FSL task to present only five objects per session (the “small-set FSL task”; Fig. 7A). The task (Fig. 7B) proceeded as in experiment 3, except that (i) each problem continued until the completion of five or six correct trials; (ii) in each problem, two objects (S^+^ and S^−^) were randomly selected from the five session-specific objects (Fig. 7A, bottom right); and (iii) on each trial, S^+^ and S^−^ were placed at two of three possible locations (90°, 210°, and 330°; Fig. 7A, top right). In separate blocks, we recorded neural activity during the MGS task (Materials and Methods). We recorded activity from 192 FPC and 213 pos-PFC neurons in monkeys K and H (Fig. 1K and table S1)

Both monkeys performed significantly above chance (50%) from the second trial after problem change, indicating rapid learning of new S-R relations (Fig. 7C, one-sample *t* test). We calculated population PEV time series using a three-way ANOVA with factors: ordinal trial position from problem change (six levels: first to fifth, and trials sixth and later combined), chosen object (five levels), and chosen location (three levels) (Fig. 7, D and E). Correct and error trials were analyzed together, except during the perireward period (green-shaded area), where only the first to sixth correct trials after problem change were analyzed. In both FPC and pos-PFC, the PEV for the critical interaction term (chosen object × ordinal trial position) did not significantly increase relative to noise at any point (Fig. 7E, leftmost panel, one-sample permutation test, FDR-corrected), indicating that selectivity for chosen object did not differ between exploration and exploitation phases. Thus, unlike the lateral orbitofrontal cortex (*29*) and anterior cingulate cortex (*30*), neither FPC nor pos-PFC contributed significantly to credit assignment, a key process in learning new S-R relationships.

Notably, significant PEV for the main effect of ordinal trial position appeared only in the pos-PFC (Fig. 7D, leftmost panel), emerging well before array onset (arrow) and disappearing by 480 ms after. This contrasted markedly from experiment 3, where significant PEV for this factor emerged only after array onset, in both the FPC and pos-PFC (Fig. 5C, middle). Additional analyses revealed that, in the pos-PFC, this pre–array onset ordinal-trial-position selectivity was primarily driven by an increase in positive-slope neurons signaling the occurrence of familiar contexts (Fig. 7, F and G). Specifically, in the two prearray analysis time windows (−0.7 to −0.35 s and −0.35 to 0 s from array onset; Fig. 7G), the percentages of positive-slope neurons in the pos-PFC (magenta; 10.8 and 8.9%, respectively) significantly exceeded chance (2.5%) (*P* = 0.004 and *P* = 0.03, *P* values corrected as in Fig. 5E). However, in the 0- to 0.35-s window after array onset, the percentages of negative- and positive-slope neurons in the pos-PFC reversed (8.5 and 6.6%, respectively), both matching the result from epoch B in experiment 3 (Fig. 5E; 7.2 and 8.7%, respectively; *P* > 0.39 for both across-experiment comparisons; Fisher’s exact test). These results indicate that the pos-PFC activity flexibly adjusted when and how ordinal-trial-position information was expressed during the trial despite the large difference in object set size: In experiment 3, ordinal-trial-position selectivity emerged mainly after array onset, whereas in experiment 4, it emerged well before array onset (driven primarily by positive-slope neurons) and then transitioned to the post–array onset pattern comparable to experiment 3. In contrast, the FPC showed no such adjustment, suggesting a limited role in tracking learning progress. The learning-related activity modulations in the pos-PFC observed here have precedents in the literature (*31*, *32*). In working memory tasks, overall LPFC firing rates have been reported to decrease or increase (depending on task complexity) as task learning progresses. Although those studies focused on learning task rules rather than object identity per se, together these findings suggest that posterior LPFC contains mechanisms that can continuously adjust neural activity as learning unfolds.

Another key observation was the significant difference in PEV for the factor chosen object between the two regions (Fig. 7D, middle). Near reward time, the pos-PFC showed a robust increase in object selectivity, suggesting that it closely monitored the chosen object’s identity. By contrast, the FPC weakly represented chosen object identity, only occasionally reaching significance and to a significantly lesser degree than the pos-PFC. In addition, only the pos-PFC exhibited significant PEV for the interaction term (chosen object × chosen location) (Fig. 7E, rightmost panel), representing “what in where” information (*33*) throughout the trial, with two prominent peaks after array onset and target acquisition. In contrast, even in a task where only object information was relevant, the FPC’s strongest representation near reward time focused only on chosen location (Fig. 7D, rightmost panel). This suggests that, regardless of behavioral context, the monkey FPC carries only limited object information. Since learning in natural settings cannot rely solely on spatial information, these results suggest that, although the monkey FPC may participate in rapid learning, it carries a narrower range of learning-relevant information than pos-PFC and is unlikely to be the principal locus for rapid learning, whether in novel contexts or involving reversals.

### Experiment 5: Testing FPC’s role in self-generated decision monitoring

Thus far, regardless of task demands, whether emphasizing spatial or object information, or involving functions previously implicated in the monkey FPC, the FPC has primarily and inflexibly engaged in retrospective coding of the chosen location associated with behavioral reports. This contrasts with the proposed role of the monkey FPC in decision monitoring, as suggested by Tsujimoto *et al.* (*11*). In their spatial CS task, on each trial, monkeys had to identify a rewarded location (RL), left or right, by integrating memory of the previous trial’s RL with a strategy cue shown at trial onset that signaled whether the RL would stay (a “stay” trial) or shift (a “shift” trial). They termed this a “self-generated” decision because it relied on internal memory of the RL. After such decisions (saccadic choice), they reported that FPC neurons exhibited significant chosen-location selectivity, sustained until both standard (0.5 s after target acquisition) and delayed (1.0 s after acquisition) reward timings. Notably, after externally instructed action selection in the MGS (control condition), where RL memory carry-over across trials was unnecessary, significant chosen-location selectivity lasted only until the standard reward but diminished before the delayed reward, just 0.5 s later. On the basis of this duration difference, the authors interpreted the FPC’s perireward activity as reflecting a monitoring signal of self-generated decisions, integrating internal and external information to promote adaptive behavioral control, rather than merely encoding the chosen location of just-executed actions.

**Fig. 8.**
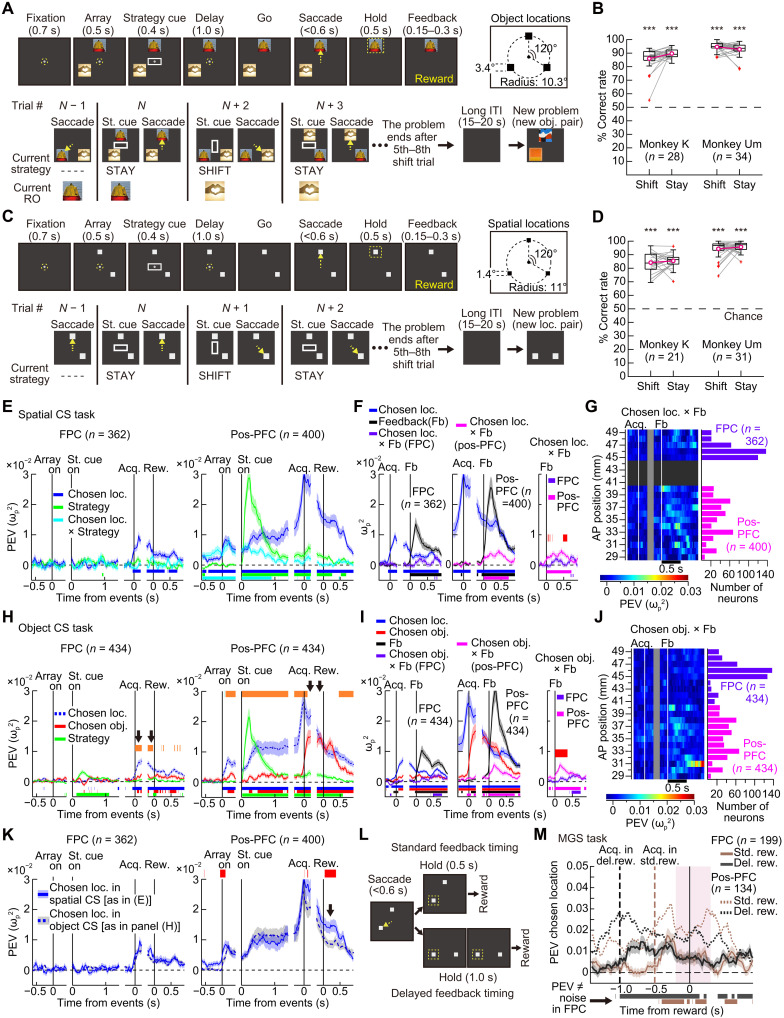
Neural activity related to spatial and object self-generated decision-making across the anteroposterior LPFC. (**A**) Object CS task. Top: Trial sequence. Bottom: Example task progression (correct only). (**B**) Behavioral performance in object CS task. (**C** and **D**) Same as (A) and (B), but for spatial CS. (**E**) Spatial CS task: Population PEV (mean ± SEM) for chosen location (blue), strategy (green), and interaction (cyan) in FPC (left) and pos-PFC (right), aligned to array onset, strategy-cue onset (st. cue), target acquisition, and reward. Lower colored bars mark significant PEV. (**F**) Spatial CS task: Population PEV for chosen location (blue), feedback (black), and their interaction in FPC (left) and pos-PFC (middle), aligned to acquisition and feedback onset (fb.). Lower bars mark significant PEV. Rightmost panel: Interaction PEV (chosen location × feedback) in FPC (purple) versus pos-PFC (magenta); upper red bars mark significant between-area differences. (**G**) Spatial CS task: Interaction PEV (chosen location × feedback) across AP locations. (**H**) Object CS task: Population PEV for chosen location (dotted blue), chosen object (red), and strategy (green) in FPC (left) and pos-PFC (right). Upper orange bars mark significant differences between location and object PEV. (**I**) Object CS task: As in (F), but for chosen location (blue), chosen object (red), feedback (black), and critical interaction term (chosen object × feedback). (**J**) Object CS task: As in (G), but for interaction (chosen object × feedback). (**K**) Chosen-location PEV in spatial versus object tasks. Upper red bars mark significant task differences. (**L**) Task progression after correct saccade under standard and delayed reward conditions. (**M**) MGS task. PEV for chosen (cue) location aligned to reward delivery in FPC (solid lines) and pos-PFC (dashed). Lower bars indicate periods when FPC PEV significantly differed from noise for standard (brown) and delayed (black) reward conditions. Pink shading, perireward period (−0.2 to 0.3 s).

To resolve this discrepancy, we introduced a novel object-based CS task in which decisions were decoupled from space (Fig. 8A). In this task, the monkey tracked the identity of a rewarded object (RO) and used a strategy cue to determine the RO on each trial (see Materials and Methods for task details). If FPC activity truly monitors self-generated decisions and evaluates their outcome, its perireward activity should be selective to the chosen object identity rather than its location. For comparison, we used the spatial CS task having the same temporal structure (Fig. 8C) in which the monkeys tracked a rewarded location (RL) among two placeholders [RL and nonrewarded location (NL)]. The spatial and object CS tasks, along with the MGS task were performed in alternating blocks (counterbalanced across sessions). We recorded activity from 362 FPC and 400 pos-PFC neurons during all three tasks, and 72 FPC and 34 pos-PFC neurons only during the object CS and MGS tasks (recording locations; Fig. 1L). Both monkeys performed significantly above chance in both shift and stay trials of the object CS (Fig. 8B; *P* < 7.6 × 10^−6^) and spatial CS tasks (Fig. 8D; *P* < 1.2 × 10^−4^).

We first examined population PEV in the spatial CS task using a two-way ANOVA (chosen location: 90°, 210°, 330°; strategy: stay/shift; Fig. 8E). The results resembled prior studies (*11*, *34*). In the FPC (left), the PEV for chosen location (blue) was significantly elevated only during the perireward period (after target acquisition), with all other factors remained nonsignificant throughout the trial. Critically, in the object CS task (Fig. 8H), a three-way ANOVA (chosen object × chosen location × strategy) showed that, during the perireward period, FPC activity (left) encoded the chosen location (blue) significantly more strongly than the chosen object (red) (arrows). Despite the task’s emphasis on object information, the chosen object PEV was weak and only sporadically significant, indicating that contrary to previous proposals, FPC perireward activity does not monitor the content of self-generated decisions but instead persistently encodes the chosen location retrospectively. In contrast, in the pos-PFC (Fig. 8H, right), the chosen object PEV increased drastically, nearly matching that for chosen location, as evidenced by a nonsignificant difference between the two measures after target acquisition (arrows).

For the 362 FPC and 400 pos-PFC neurons recorded during both CS tasks, only in the pos-PFC did the perireward PEV for chosen location significantly decrease in the object CS task compared with the spatial CS task (arrow, Fig. 8K, right). This indicates that, in the object CS task, pos-PFC activity flexibly adapted to the change in task requirements, down-weighting the chosen-location signal and reallocating processing resources to encode the chosen object after target acquisition. In contrast, FPC activity lacked such flexibility and consistently encoded chosen location with equal strength regardless of task-relevant modality. In addition, unlike the pos-PFC (fig. S8A, right), the FPC (left) showed no significant PEV for interaction terms throughout the object CS trial, indicating little to no capacity for integrating object information during object-based self-generated decisions.

Next, to assess FPC’s role in decision outcome evaluation after feedback, we analyzed correct and error trials to determine if FPC activity discriminated RL versus NL in the spatial CS task (Fig. 8F, two-way ANOVA: chosen location × feedback) and RO versus nonrewarded object (NO) in the object CS task (Fig. 8I, three-way ANOVA: chosen object × chosen location × feedback). For both critical interaction terms, [chosen location × feedback] in the spatial CS task and [chosen object × feedback] in the object CS task (rightmost panels), the FPC (purple) exhibited only a weak, delayed rise in PEV after feedback, whereas the pos-PFC (magenta) showed an earlier and significantly stronger increase. A segmental analysis along the AP axis confirmed these results (Fig. 8, G and J). Furthermore, in the object CS task, the three-way interaction PEV also rose earlier and significantly more strongly in the pos-PFC than in the FPC (fig. S8B). These findings indicate that the pos-PFC robustly and promptly evaluates decision outcomes across both spatial and object domains, while the FPC’s involvement is minimal and delayed, likely reflecting inputs from the pos-PFC or other regions.

Returning to experiment 4 (Fig. 7), we examined whether the same phenomena occurred during the feedback period in the small-set FSL task in which monkeys evaluated whether the chosen object was an S^+^ or not. Again, the PEV for the interaction term [chosen object × feedback] in the pos-PFC rose earlier and significantly more strongly than in the FPC whose increase was negligible (fig. S9). These results further emphasize the pos-PFC’s dominant role in decision evaluation across tasks and modalities, compared with the FPC.

Last, in separate sessions (*n* = 23, monkey Um), following the prior study (*11*), we introduced a delayed feedback condition (1.0 s after acquisition) for both the spatial CS and MGS tasks (Fig. 8L and fig. S10; 199 FPC and 134 pos-PFC neurons recorded in both tasks). To match the spatial CS task, the MGS task was slightly modified by adding placeholders (fig. S10A, bottom row). Contrary to the prior study’s conclusion that FPC’s prolonged (up to 1 s) chosen-location selectivity during the perireward period was exclusive to the spatial CS task, we found significant chosen-location selectivity in the FPC (and pos-PFC) continuing until the delayed reward (up to 1 s) in both MGS and spatial CS tasks (Fig. 8M for MGS; for detailed results in both tasks, including representative single-unit data, see fig. S10). Note that the prior study’s conclusion was based on 34 FPC neurons, used to assess whether the chosen-location selectivity persisted until the delayed reward in the MGS task, with just 8 recorded in both tasks contributing to the time-course analysis [figure 7, c and d, in (*11*)]. Furthermore, the MGS task in experiment 1 (Fig. 2) also included both standard (250 ms) and delayed (600 ms) feedback conditions. Despite not involving self-generated decision-making, we again found in the MGS, that significant chosen-location selectivity in the FPC (and in the pos-PFC) persisted until the delayed reward (737 FPC and 736 pos-PFC neurons recorded in both reward conditions; fig. S11). These results suggest that the duration of significant chosen-location selectivity after the behavioral response is not a useful metric for evaluating FPC’s role in monitoring self-generated decisions; instead, FPC’s perifeedback activity predominantly encodes retrospectively the action just taken, largely independent of task context.

### Monkey FPC activity exhibits characteristics of the task-negative network

We have demonstrated that key functions previously attributed to the monkey FPC are more robustly represented in the posterior-to-mid LPFC. In contrast, FPC showed little flexibility in adapting to changing environmental demands, encoding primarily and inflexibly the chosen location of just-executed actions. This task-indifferent nature led us to hypothesize that the monkey FPC is largely disconnected from the task-positive, cognitive control network. To test this, we examined whether overall firing rates in the FPC and pos-PFC, averaged across all conditions, showed significant task-related (excitatory) activation or deactivation relative to baseline during each of the four tasks (the MGS, attention, FSL, and CS tasks; see Materials and Methods for analysis database). Using cluster-based paired permutation test, we compared population overall firing rates during baseline (final 0.6 s of the fixation period) against those in successive 50-ms bins across the period of active task engagement, defined as the interval from the final cue (when all instructions were presented) until reward, during which the monkey performed internal computations without further stimulus presentation.

**Fig. 9.**
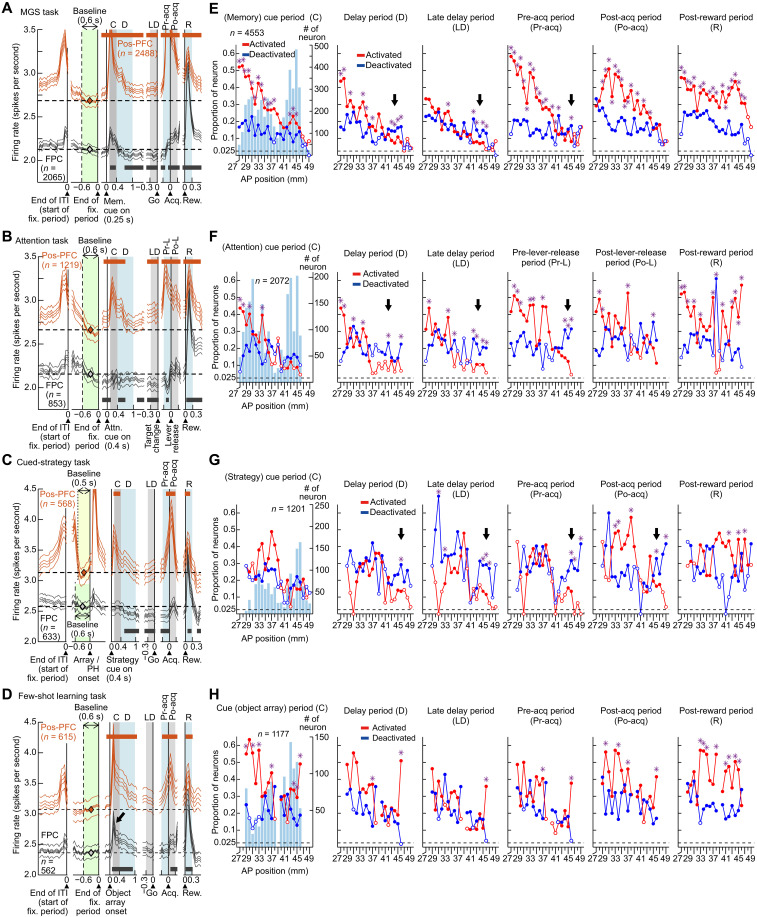
Anteriorly enhanced task-related deactivation in the LPFC across multiple tasks. (**A**) MGS task. Population-averaged overall firing rate from 1.0 s before the end of ITI to 0.7 s after reward in pos-PFC (orange) and FPC (gray), aligned to ITI end, end of fixation period, cue onset, go signal, target acquisition, and reward delivery. Green shading marks baseline (final 0.6 s of fixation period). Diamonds (with dashed lines) indicate the mean baseline firing rate in pos-PFC (orange) and FPC (gray). Orange and gray horizontal bars indicate bins significantly different from baseline (cluster-based paired-permutation test, *P* < 0.05). Inner and outer error bars show within- and between-subject SEM. Gray and cyan shading indicate analysis epochs in (E): C, cue; D, delay; LD, late delay; Pr-acq, pre-acquisition; Po-acq, post-acquisition; and R, reward. (**B**) Attention task. Conventions as (A). Pr-L, pre-lever release; Po-L, post-lever release. (**C**) CS task (spatial and object versions combined). Conventions as (A), with C denoting strategy cue period. Object array and placeholder onset (“Array/PH onset”) coincided with the end of fixation period. In pos-PFC, baseline (yellow shading) was set as −0.5 to 0 s relative to the end of fixation period to minimize the influence of FP onset and ensuing saccades. (**D**) FSL task. C denotes the 0.1- to 0.4-s window after object-array onset. (**E**) MGS task. Proportion of significantly activated (red) and deactivated (blue) neurons relative to baseline across AP locations for six task periods (cue, delay, late delay, pre-acq, post-acq, and reward). Filled circles indicate proportions significantly different from chance (2.5%; FDR-corrected across AP bins); white-centered circles indicate nonsignificance. Purple asterisks mark bins where activation and deactivation proportions significantly differed. In the leftmost panel, the histogram (right *y* axis) shows the number of neurons per AP bin. (**F** to **H**) Same as (E), but for attention (F), CS (G), and FSL (H) tasks.

We observed two key findings. First, during the mid-phase of the trial [from the delay period (D) through the behavioral response (saccade or lever release); Fig. 9, A to D], the pos-PFC (orange curves) predominantly showed significant task-related activation, whereas the FPC (gray) displayed significant task-related deactivation in all tasks except FSL (see fig. S12, C and D, and note S4 for a likely explanation of this exception related to strong cue-period visual drive; arrow in Fig. 9D). Notably, despite modest per-bin effect sizes of FPC deactivation (paired-samples Cohen’s *d_z_* ≈ 0.1 to 0.2; fig. S13), this effect formed a temporally sustained significant cluster (*P* < 0.05; cluster-based paired permutation test, Fig. 9, A to C), indicating a reliable low-amplitude but prolonged suppression. This marked contrast between the two regions suggests that the pos-PFC is part of the task-positive network, while the FPC belongs to the task-negative network or is at least detached from the task-positive network. Second, both areas exhibited pronounced phasic activations after reward, with nearly identical strength and latency, suggesting a common input from the brain’s reward system. Note that in all tasks, the phasic activation near the end of ITI corresponded to the onset of FP and the monkey’s saccade toward it.

To complement this analysis, for each 1-mm AP segment, we calculated the proportion of neurons whose overall firing rate showed significant task-related activation or deactivation relative to baseline (paired-permutation test, *P* < 0.05) in each of six task periods: cue (labeled as C in Fig. 9, A to D), delay (D), late delay (LD), pre-acq (Pr-acq), post-acq (Po-acq), and reward period (R) (see Materials and Methods). We found that in the posterior-to-mid LPFC (< ∼AP 42 mm), the proportion of activated neurons (red) tended to significantly exceed that of deactivated neurons (blue) across all task periods (Fisher’s exact test, *P* < 0.05, FDR-corrected; Fig. 9, E to G). This proportion of activated neurons showed an anteriorly decreasing trend. Notably, during the delay, late delay, and pre-acq (or pre-lever) periods, at more anterior locations (> ∼AP 42 mm; arrows, Fig. 9, E to G), the percentage of deactivated neurons (blue) significantly exceeded that of activated neurons (red) in all tasks except for the object FSL task (Fig. 9H). Last, during the reward period (rightmost panel), activated neurons predominated across all AP locations in all tasks. In the CS task, a separate analysis of spatial and object CS tasks showed similar patterns across tasks (fig. S12, A, B, E, and F), confirming the reproducibility of the present findings. These results suggest that, during active task engagement, the monkey FPC not only encodes minimal task-related information but also generally shows task-related deactivation, a pattern suggestive of a task-negative or DMN function. In contrast, the posterior-to-mid LPFC exhibits task-related activation and is consistently involved in multiple cognitive demands, suggestive of the task-positive, cognitive control region (*6*, *35*). Overall, these results point to a posterior-to-anterior transition from a task-positive to a task-negative region in the monkey LPFC.

## DISCUSSION

The monkey FPC has been commonly viewed as central to adaptive behavioral control, particularly in complex, nonroutine environments. However, this notion has been shaped largely by limited experimental conditions, as well as the absence of direct comparisons with other prefrontal areas. Here, by extensively sampling neural activity throughout the anteroposterior LPFC, we fundamentally revised the FPC’s role within the broader LPFC. Anteriorly, neural engagement progressively weakened and was weakest in the FPC itself, though not absent, for cognitive orchestration, rapid novel learning, and self-generated decision monitoring, functions that have previously been considered core to the leading role of the monkey FPC in adaptive control. The FPC’s perifeedback activity, previously proposed to integrate self-generated and environmental information and thus implicated in learning, was notably inflexible, predominantly encoding the chosen location of the just-executed action regardless of task demands, suggesting a limited role in flexible learning-related updating. This rigidity was in stark contrast to the flexibility of the posterior-to-mid LPFC, whose activity dynamically adjusted to multiple cognitive demands. In novel learning and self-generated decision monitoring, we first replicated several observations from earlier FPC studies, validating our experimental approach. Nevertheless, by incorporating novel tasks and expanding LPFC sampling, we gained a more comprehensive perspective on the role of the FPC relative to the broader LPFC. We also observed anteriorly intensified task-related deactivation extending into the FPC. Together, these findings reveal a reversed functional gradient in the monkey LPFC, characterized by posterior dominance and frontopolar task-related deactivation, which challenges prevailing anterior-dominant models of LPFC function across primates. Experiments 1 to 5 are discussed in detail in note S1.

Overall, our findings align with a line of anatomical evidence suggesting that the monkey FPC is not directly analogous to its human counterpart, a point often overlooked in discussions emphasizing cross-species similarities in prefrontal organization (*3*, *36*). For example, (i) the human FPC is disproportionately expanded compared with those in apes and monkeys (*37*, *38*); (ii) the monkey anterior LPFC lacks three major sulci present in the human lateral FPC (*39*), suggesting the absence of a region analogous to the human lateral FPC, (iii) resting-state functional MRI (fMRI) indicates that the functional connectivity of the human lateral FPC more closely resembles the mid-dorsolateral PFC (mid-dlPFC) than the FPC of the macaque (*40*); and (iv) a tract-tracing study (*41*) suggested that the monkey FPC corresponds predominantly to the medial portion of the human FPC that is subdivided into medial, orbital, and lateral aspects. Furthermore, a meta-analysis of 148 tract-tracing studies places the monkey FPC relatively low in the prefrontal hierarchy, on the basis of efferent-afferent and laminar-based connection asymmetries (*42*). Our present findings that the monkey FPC (i) predominantly encodes action selection with highly limited object selectivity and minimal involvement in object-based decision-making, (ii) contains a moderate but significant proportion of neurons encoding reward-prediction-error–related signals, and (iii) exhibits default mode–like task-related deactivation during externally directed behavior, suggest that the monkey FPC is an extension of the medial PFC that shares these characteristics (*30*, *43*–*45*).

Regarding task-related deactivation in the FPC (Fig. 9), we identified a boundary at approximately AP 41 to 43 mm that marks the turning point in a gradual posterior-to-anterior transition from a task-positive to a task-negative region (Fig. 9, E to G). A previous monkey positron emission tomography study using working memory tasks reported significant task-related deactivation, a potential DMN hub, in the mid-dlPFC and medial area 10, along with the well-known medial frontoparietal regions (*43*). Similarly, an fMRI meta-analysis using 15 visual tasks found task-related deactivation in the mid-dlPFC (but not in the FPC) (*46*), with deactivation peaks in F99 space corresponding to approximately AP 38 to 40 mm in our stereotaxic space (*44*). [One possible reason why this fMRI meta-analysis did not detect FPC deactivation is the difficulty of imaging the frontal tip of the brain without specialized scanning sequences (*47*).] In the present study, the consistent anteriorly enhanced task-related deactivation provides the first electrophysiological evidence that the anterior LPFC may participate in a task-negative network. This notion is further reinforced by our finding that, across the full AP extent of the LPFC, this anteriorly enhanced deactivation coincided with a progressive decrease in task-related selectivity (and neural flexibility), a hallmark of detachment from a cognitive control (task-positive) network. This anterior decrease in task-related selectivity mirrors previous reports that, within the posterior half of the LPFC, neural selectivity for abstract rules, rewarding objects, and rewarding locations diminished anteriorly (*48*–*50*).

In quantifying task-related activation and deactivation, we used a standardized baseline period to ensure consistency across tasks and recording areas. We nevertheless acknowledge that the choice of baseline is important, as different reference periods can influence the apparent magnitude of activation or deactivation. For example, if baseline were taken at the very end of the fixation period, when firing rates are already near their minimum, FPC activity during subsequent task periods might not appear strongly suppressed. However, such a choice would ignore the possibility that the gradual decrease in FPC firing rate during the fixation period already marks the subject’s entry into the task, i.e., a preparatory, task-related deactivation. For this reason, we also present population firing-rate time courses spanning the entire trial for all four tasks (Fig. 9, A to D). This presentation reveals a robust qualitative dissociation between regions: Posterior LPFC exhibits prominent event-related increases followed by sustained elevations around major task events, whereas in the FPC, firing rates tend to show a gradual decline across the trial in three of the four tasks (Fig. 9, A to C). We consider this marked difference in temporal profile over the course of the trial to be a key observation supporting task-related deactivation, independent of any particular baseline choice. At first glance, the magnitude of FPC deactivation may appear modest. Because task-related deactivation reflects a state-like, induced change in ongoing activity rather than a response tightly time-locked to discrete task events, modest pointwise effect sizes are expected. The critical feature is sustained, directionally consistent suppression, which we observe in the FPC.

We acknowledge several limitations of the present study. First, our interpretation of the macaque FPC as a default mode–like region does not imply that it is entirely devoid of control-related function. For example, although our results in experiments 1 and 2 suggest a limited role for FPC and a greater role for posterior PFC in resource allocation, prior lesion work has been interpreted as suggesting an essential contribution of FPC to this function [(*8*); see also note S1 for discussion of alternative interpretations of those lesion findings]. How these lesion-based interpretations relate to the present physiological findings remains to be clarified. More generally, given how little is still known about the monkey FPC, a key lesson from the present work is that putative FPC functions should be evaluated through direct, region-by-region comparison with other prefrontal areas, an approach that has proven particularly informative in human studies (*51*), rather than being inferred from FPC-only data in isolation. Second, the extent to which the macaque FPC corresponds to a DMN region remains unresolved. Although we observed a temporally sustained task-related deactivation in the FPC, further work will be required to clarify the relationship between this tonic suppression during task engagement and established DMN signatures (*52*). It is worth noting that the same FPC sites nonetheless exhibited detectable task-related signals, including location coding, robust reward responses, and, while weaker than in posterior LPFC, nonzero learning–related modulation. Notably, however, the presence of task content–selective neuronal activity does not by itself preclude DMN membership, as the co-occurrence of task content–selective responses and task-related deactivation has been reported in macaque posterior cingulate cortex, which has been implicated as part of the primate DMN (*52*, *53*). These observations raise the possibility that the macaque FPC may constitute an interface zone between the cognitive-control regions of mid-to-posterior LPFC and the medial frontoparietal DMN core.

In summary, our findings challenge the view that the monkey FPC plays a central role in adaptive behavioral control in complex, nonroutine environments and instead highlight the dominant contribution of posterior LPFC to functions previously attributed to the FPC. This observation raises broader questions regarding the conservation of prefrontal organization across primate species (*54*). Specifically, we have demonstrated that the monkey LPFC exhibits a posterior-dominant functional gradient, reversing the anterior-dominant pattern widely assumed for humans. Consequently, findings from the monkey FPC may have limited explanatory power for understanding human prefrontal organization, particularly in its lateral-anterior extent. The marked anatomical and functional expansion of the human FPC, supporting uniquely human cognitive capacities such as mental simulation (*55*), self-inference (*1*), and hierarchical reasoning (*22*), may represent an evolutionary addition beyond the explanatory scope of macaque models.

## MATERIALS AND METHODS

### Subjects and apparatus

Experiments 1 to 5 were performed in four adult female macaques: two Japanese macaques (*Macaca fuscata*; monkeys K and Um; 8.4 kg, 7 years old and 7.5 kg, 11 years old, respectively) and two rhesus monkeys (*Macaca mulatta*, monkeys T and H; 4.6 kg, 9 years old and 6.0 kg, 11 years old, respectively; ages as of the start of the recording sessions). Different subsets of two to three monkeys participated in each experiment: experiments 1 and 2 (K and T); experiment 3 (K, H, and Um); experiment 4 (K and H); experiment 5 (K and Um). All monkeys were housed individually under a 12-hour light/dark cycle (light: 8:30 a.m. to 8:30 p.m.). Before this study, monkey K had participated in two other posterior LPFC recording studies, which addressed research objectives distinctly different from those of the present investigation (*20*, *56*).

During the experiments, the monkeys sat quietly in a primate chair within a dark, sound-attenuated shielded room. Their head movements were noninvasively restrained using a thermoplastic head cap made of standard splint material (MT-APU, 3.2 mm thick, CIVCO Radiotherapy, IA) (*20*, *57*, *58*). Visual stimuli were presented on a 17-in. (43-cm) thin-film transistor monitor positioned 50 cm from the monkey’s eyes. Eye movements were sampled at 120 Hz using an infrared eye-tracking system (ETL-200, ISCAN, MA). Fixation to the FP was controlled within a 6.5° square window (visual angle). Behavioral tasks were controlled by the TEMPO system (Reflective Computing, WA). The visual object stimuli used in experiments 2 to 5 were obtained from the online gallery of the Art Institute of Chicago and the Open Images database (*59*) and were cropped to the appropriate size. All experimental procedures were approved by the Animal Research Committee at the Graduate School of Frontier Biosciences, University of Osaka (approval no. FBS-23-003) and complied fully with the guidelines of the National BioResource Project “Japanese Macaques.”

### Implantation of recording chambers

Under general anesthesia and aseptic conditions, plastic recording chambers were stereotaxically implanted onto the lateral surface of the prefrontal cortex, guided by structural MRI (Fig. 1). In monkey H (Fig. 1G), a single cuboid chamber (internal dimensions: 12.0-mm width by 16.0-mm depth by 15.0-mm height; S-Company Ltd., Tokyo) was implanted, covering the FPC and extending posteriorly into the mid-to-posterior LPFC. For the remaining three monkeys, we implanted two chambers positioned along the AP axis: an anterior chamber over the FPC and a posterior chamber over the mid-to-posterior LPFC (pos-PFC; Fig. 1A). The design of the FPC chamber varied among monkeys. For monkeys K (right hemisphere) and Um, a cuboid chamber was used (internal dimensions: 9.0 by 12.0 by 15.0 mm; S-Company Ltd). For monkey T, a cylindrical chamber was used (internal diameter, 8.0 mm; height, 18.0 mm). For the left hemisphere of monkey K, an elliptical cylindrical chamber was used (internal major/minor axes, 11.0/8.0 mm; height, 18.0 mm; O’hara & Co., Ltd., Tokyo). The design of the pos-PFC chamber also varied. Monkeys K (both hemispheres) and Um received cuboid chambers (internal dimensions: 12.0 by 16.0 by 15.0 mm; S-Company Ltd). For monkey T, we implanted a cylindrical chamber (internal diameter: 12.7 mm; product no. RC-T-S-P; Gray Matter Research, MT). In total, neural recordings were obtained from five hemispheres across four monkeys (Fig. 1, C to G).

For surgical planning and identifying recording locations, we acquired high-resolution T1-weighted anatomical images in each monkey multiple times before and after chamber implantation under general anesthesia [medetomidine: 0.03 mg/kg, intramuscularly (IM); midazolam: 0.2 mg/kg, IM; butorphanol tartrate: 0.3 mg/kg, IM]. MRI data were obtained using a 3T Magnetom Prisma-Fit MRI scanner (Siemens, München, Germany) with a custom-built 12-channel phased-array receiver coil (Takashima Seisakusho Co., Ltd.). Preoperative anatomical scans typically used a 0.67-mm isotropic voxel size, while a 0.4-mm isotropic voxel size was used for identifying recording locations. Multiple anatomical scans were collected for each monkey as long as anesthesia remained effective, and the datasets were averaged to improve the signal-to-noise ratio (SNR). We reconstructed the monkey brains and recording sites using 3D Slicer software (version 5) (*60*). Each monkey’s brain was semiautomatically rendered in three dimensions. Electrode tracks and their contact points on the brain surface were determined by aligning structural MRI images with the trajectory of the corresponding grid hole through which each track passed (Fig. 1B). Coordinates for each contact point were determined using standard stereotaxic coordinates aligned to the orbitomeatal plane and expressed on the AP and ML axes relative to the interaural midpoint (origin).

### Neural recording

We recorded all neurons encountered without any preselection. Across all monkeys and experiments, the FPC chamber yielded 140 unique sites in 155 total insertions [red and purple circles in Fig. 1 (C to G); AP coordinates ranged from 39.9 to 49.0 mm], while the pos-PFC chamber yielded 151 unique sites in 158 insertions (green circles; AP range, 26.5 to 40.8 mm).

For pos-PFC recordings in monkey T only (experiment 1; Fig. 1F, green dots), we used a unique setup consisting of a 32-channel semichronic microdrive system (SC-32, Gray Matter Research). This system housed 32 single-contact tungsten electrodes arranged in a grid pattern with an interelectrode spacing of 1.5 mm. Approximately 3 hours before each session, monkey T was briefly transferred to the testing room, and each electrode was advanced individually by at least 65 μm (typically 130 μm) to ensure the isolation of new neurons. For all other chambers, we instead used 24- or 32-channel acute linear multielectrode probes (U-Probe, Plexon, TX) with an interelectrode spacing of 150 μm along a single shaft. Typically, a single U-Probe was inserted into each chamber using a custom-made grid designed for each chamber (φ0.6-mm holes, 1-mm grid spacing). To advance the U-Probes, we used an ultracompact micromanipulator (MO-903, Narishige, Tokyo) or a custom-built compact hydraulic microdrive (S-Company Ltd.). As in our previous studies (*20*, *56*), we first punctured the dura using a guide tube (a shortened 24-gauge needle), then advanced the U-Probe into the cortex slowly in incremental steps, typically 500 μm at a time. The cortical surface was identified by monitoring pulsatile fluctuations (electrocardiogram) on superficial electrodes, and typically three to five superficial channels remained above the cortical surface during recordings. After insertion, we allowed 1 to 1.5 hours for recordings to stabilize, during which monkeys remained seated, watched nature and animal video clips, and received small snacks.

To ensure that differences in neuronal responses across recording locations were not due to systematic variations in signal processing or spike detection, we applied identical procedures to all recording chambers and monkeys. Raw extracellular neural signals were amplified and recorded using an RZ2 Bioamp Processor (Tucker-Davis Technologies, FL). Task-event information and eye-movement data were synchronized and transmitted to the RZ2. Neural signals were sampled at 24,414.08 Hz, and behavioral data were sampled at 1017.25 Hz. Single-neuron activity was extracted by band-pass filtering the raw signals (300 Hz to 6 kHz), followed by semiautomatic offline spike sorting using Offline Sorter software (Plexon). During recording, action potentials were flagged for offline sorting if their waveforms’ trough or peak, typically the trough due to the extracellular recording method, crossed a threshold set at ±3.5 times the SD of the raw signal’s continuously updated root mean square value. This uniform cutoff method ensured a consistent SNR across all recording sites.

To confirm that the SNR was comparable across recording areas, we compared waveforms from all isolated single units recorded in the FPC and pos-PFC chambers using a standard method (*61*). For each neuron, SNR was defined as the peak-to-trough amplitude of the mean waveform divided by the SD of baseline noise, computed from the first 0.12 ms (i.e., the initial three data points before depolarization) of each waveform. Across experiments 1 to 5, we isolated 2264 single units in the FPC and 2622 in the pos-PFC. Of these, 2260 units (99.8%) in the FPC and 2607 units (99.4%) in the pos-PFC met the criterion of SNR > 4, indicating excellent isolation quality in both recording locations.

For action-potential detection and unit inclusion, we applied a uniform refractory-period criterion, using a minimum interspike interval of 1 ms (1000 μs) and permitting no refractory-period violations. Most neuron-yielding channels contained only one well-isolated unit, with the proportion of neurons recorded as a single unit on a given channel being 75% (experiment 1), 83% (experiment 2), 80% (experiment 3), 84% (experiment 4), and 82% (experiment 5).

### Statistical analysis

In this study, all population-level analyses included all recorded neurons without any prescreening procedure based on neuron’s activity patterns, except in the population histograms in Fig. 6D, which included only neurons meeting specific criteria. All statistical tests were two tailed and conducted in MATLAB (MathWorks). To address inflated type I error rates in multiple hypothesis testing, we applied either the Benjamini-Hochberg (BH) procedure (*62*) to control the FDR or the Holm’s sequentially rejective Bonferroni (Holm-Bonferroni) procedure, as appropriate. Corrected *P* values are reported unless otherwise noted.

### Behavioral tasks and analysis

Details of behavioral tasks and analyses for each experiment are described primarily in the main text, with additional information provided below.

#### Experiment 1

Monkey T completed 155 ± 34 single-task MGS and 184 ± 29 dual-task MGS correct trials per session, whereas monkey K completed 143 ± 24 single-task MGS and 176 ± 37 dual-task MGS correct trials (mean ± SD). In the attention task, a custom-made lever for behavioral responses was attached to the front of the chair at the monkey’s chest height. The lever remained in place during the dual-task MGS blocks but was removed during the single-task MGS blocks, as it was unnecessary for MGS performance alone and to avoid unintended associations. In dual-task MGS trials, because our primary focus was to examine how MGS-related activity differed between single- and dual-task conditions, we ensured clear temporal separation of MGS-related activity from attention task–related activity by inserting the memory cue at least 0.7 s away from any attention task event (catch change or target change) and always before a target change (Fig. 2D). Within these constraints, we randomly selected timings of memory cue presentation (1.0 to 4.1 s or 1.0 to 5.0 s from the attention cue offset in short trials and long trials, respectively, of the attention task). Despite this 0.7-s gap, extending the MGS analysis window beyond it (e.g., to 1 to 2 s after memory-cue onset) sometimes led to overlap with attention task events on some trials. In these trials, only the neural activity before that attention task event was used for analysis. For the analysis of attention task performance, we assessed whether task difficulty differed among the three attention task conditions (the upper-ring, lower-ring, and fixation-ring conditions) by comparing the percent-correct rates and lever-release RTs in single attention task trials. Because we were interested in all pairwise comparisons among these conditions, we performed a series of three pairwise comparisons using a paired-permutation test with *P* values corrected by the Holm-Bonferroni procedure. There were three types of errors in the attention task: (i) fixation break error before the target change, (ii) premature lever release before the target change, and (iii) failure to initiate lever release within 0.6 s after the target change. Only the second and third types of errors were considered when calculating percent-correct rates.

To assess the impact of dual-task performance on MGS accuracy, we compared percent-correct rates between the single-task MGS condition and the three dual-task MGS conditions. Session-by-session percent-correct rates were calculated by dividing the number of correct trials by the total number of trials in which the monkeys successfully reached the go signal for the MGS (i.e., the end of the follow-up fixation period; Fig. 2D). A correct trial was defined as one that satisfied both of the following criteria: (i) successful saccadic target acquisition within 0.5 s after the go signal, and (ii) successful gaze-holding at the correct placeholder for 0.25 or 0.6 s. Any other eye movement after the go signal was considered an error (i.e., failure to acquire the correct target or failure to maintain fixation after acquisition). Because single-task MGS did not include a target change or a subsequent lever release, these events were scheduled but implemented as “empty events,” producing no changes in the stimuli and requiring no behavioral response.

For the neuronal analyses in Fig. 3 (G and I) (percentages of the dual>single location-selectivity and dual>single firing-rate patterns), we used the following time windows for each task period: fixation (final 0.6 s of the fixation period), memory cue (0.1 to 0.4 s from cue onset), delay (0.4 to 1.0 s from cue onset), and pre-go (−0.3 to 0 s from the saccade go signal). Neural recording sites in experiment 1 covered ∼20 mm along the AP axis, encompassing the full principal sulcal region and extending beyond it (AP 26.5 to 46.1 mm, stereotaxic coordinates). Along the AP axis, 47 FPC sites (red markers; Fig. 1H) spanned 6.2 mm (AP 39.9 to 46.1 mm), and 69 mid-to-posterior LPFC sites (termed pos-PFC; green markers, Fig. 1H) spanned 13.5 mm (AP 26.5 to 40.0 mm).

#### Experiment 2

During the object FSL task, each problem of this task contained two predefined secondary-task insertion events, each occurring at a randomly designated ITI of the FSL task, between the second trial and the seventh correct trial within each problem. At each designated ITI, one of the three conditions was randomly inserted: MGS insertion, time-out/free-reward insertion, or no insertion (control). The two designated ITIs were separated by at least two to three correct trials. Because the no-insertion condition involved no secondary-task event, 11% (one of nine) of problems contained no actual insertion events, 44% (four of nine) contained one, and 44% (four of nine) contained two. Thus, this design ensured that a sufficient number of trials were obtained for both the MGS-insertion and time-out/free-reward conditions. In the MGS task, there were six possible cue locations (30°, 90°, 150°, 210°, 270°, or 330°) on an imaginary circle with a 12° radius. To calculate FSL-task performance (Fig. 4E), we included all trials regardless of whether a secondary-task event occurred before or after that trial, excluding only trials aborted because of fixation breaks before the go signal. For the three-way ANOVA analyzing FSL activity (Fig. 4H), we included trial location relative to the insertion event (pre versus post) as a factor, along with insertion type and chosen location. For the pre- and post-insertion levels, neural activity was averaged across two correct trials immediately before the insertion event (−2, −1), and two correct trials immediately after it (+1, +2), respectively. For the two-way ANOVA analyzing MGS activity (Fig. 4I), 26 FPC neurons were excluded because of a code error in the first session for monkey T, which resulted in the cue location of the dual-task MGS trials not being recorded.

#### Experiment 3

In the modified FSL task, the location and size of object presentation were identical to those in experiment 2 (Fig. 4B). In Fig. 6D (normalized population histograms of negative-slope neurons), each neuron’s firing rate in successive 50-ms bins was normalized to its mean firing rate during the period from −1.0- to 1.0-s window relative to reward delivery, averaged across all trials. Because statistical comparisons between different reward conditions used a within-subject design (paired permutation test), we report within-subject SEM for the error bars (*63*, *64*). The comparison between the 1st reward (cyan) and the 8th reward (brown) is expected to yield a significant difference (1st > 8th) because the selection criterion for negative-slope neurons required a decrease in activity from the 1st to the 8th reward. Therefore, testing this contrast on the selected neurons constitutes double-dipping. The remaining three comparisons, however, are valid because they are independent of the selection criterion.

#### Experiment 4

In addition to the small-set FSL task, we recorded neural activity during the MGS task in separate blocks. The MGS task used the same parameters as in experiments 2 and 3, except that it included only three possible cue locations (90°, 210°, and 330°) rather than six, to match the object presentation locations in the small-set FSL task. For neural analyses of the small-set FSL task, the three-way ANOVA ([Ordinal trial position] × [chosen object] × [chosen location], Fig. 7, D and E) used a reduced model (main effects and two-way interactions only) rather than a full model, because the number of trials was insufficient to reliably estimate the three-way interaction.

#### Experiment 5

In the object CS task (Fig. 8A), each trial began with a 0.7-s fixation period, followed by the appearance of two objects at two of three predetermined locations (array period). A strategy cue then appeared around the FP (strategy cue period). A horizontally elongated cue (ellipse for monkey K, rectangle for monkey Um; subtending 2.3° × 5.7°) indicated that the previously RO remained rewarded (stay trial). A vertically elongated cue (rectangle with one flicker for monkey K; plain rectangle for monkey Um) indicated that the previously NO became rewarded (RO), and the previous RO became NO (shift trial). After a 1.0-s delay period, the FP disappeared (go signal), prompting the monkey to saccade to one of the two objects within 0.6 s and hold fixation for 0.5 s (hold period). The unchosen object disappeared upon selection. If the chosen object was the RO, the monkey received a reward; otherwise, the chosen object turned blue (error signal), and correction trials with the same strategy cue continued until the monkey responded correctly. To ensure learning of the new RO after a switch, each correct shift trial was always followed by a stay trial; otherwise, shift and stay trials were randomized. Trials were grouped into problems, each of which ended once five to eight shift trials were completed with the same object pair. A long ITI (15 to 20 s) then preceded the start of a new problem with a new object pair (Fig. 8A, bottom right). Each daily session introduced only five novel objects. The same object pair was not repeated in successive problems. The first trial of each problem was always a stay trial with a randomly chosen RO. Across 62 sessions, excluding correction trials, the two monkeys performed an average of 347.3 ± 79.2 trials per session (mean ± SD, including both correct and error trials), comprising 118.8 shift trials (34.2%) and 228.5 stay trials (65.8%).

The spatial CS task (Fig. 8C) had the same temporal structure as the object CS task, except that the monkeys tracked a RL among two placeholders [the RL and a nonrewarded location (NL)]. Throughout each problem (consisting of five to eight shift trials), the same pair of locations was used, selected from the 90°, 210°, and 330° locations. On each trial, choosing the RL resulted in reward delivery, whereas choosing the NL caused the selected placeholder to turn red (error signal). As in the object CS task, each correct shift trial was always followed by a stay trial. Across 52 sessions, excluding correction trials, the two monkeys performed 201.0 ± 62.5 (correct and error) trials per session, comprising 69.1 shift trials (34.4%) and 131.9 stay trials (65.6%).

For behavioral analyses of both the object and spatial CS tasks, we excluded (i) the first trial of each problem and (ii) correction trials. For neural analyses, we excluded (i), (ii), and error trials (unless otherwise noted). Thus, all trials included in the neural analysis were immediately preceded by a correctly performed trial. In the object CS task, behavioral performance was as follows: monkey K, shift trial, 86.0 ± 8.0%, stay trial, 89.7 ± 3.4%; monkey Um, shift trial, 94.6 ± 3.3%, stay trial, 92.8 ± 4.5% (mean ± SD; chance = 50%) (Fig. 8B). In the spatial CS task, performance was as follows: monkey K, shift trial, 84.2 ± 7.8%, stay trial, 85.4 ± 5.7%; monkey Um, shift trial, 94.3 ± 6.2%, stay trial, 95.7 ± 3.8% (Fig. 8D).

During the main recording sessions, the MGS task was similar to that used in experiment 4, featuring three possible cue locations (90°, 210°, and 330°). However, the temporal structure differed: fixation period = 1.2 s, memory cue period = 0.25 s, delay period = 1.15 s, response period <0.6 s, and hold period = 0.5 s. Consequently, the interval from the memory-cue onset to the go signal was 1.4 s, matching the duration from strategy-cue onset to the go signal in the CS tasks.

### Task period versus baseline firing rate analysis

In comparing the firing rate during the active task period to that of the baseline period (Fig. 9), the MGS results (Fig. 9, A and E) were derived from a combined dataset consisting of both single-task and dual-task MGS trials from experiments 1 and 2, as well as single-task MGS trials from experiments 3 to 5. However, we excluded MGS data from the supplemental portion of experiment 5 (posterior PFC, *n* = 134; FPC, *n* = 199; fig. S10), because of substantial differences in stimulus presentation compared to the standard MGS tasks (see fig. S10A, bottom row). The attention task results (Fig. 9, B and F) were derived from all attention task trials in experiment 1, including those performed as a single task and those performed in a dual-task format. The CS task results (Fig. 9, C and G) were derived from experiment 5, with spatial and object CS task data combined (for individual results, see fig. S12, A, B, E, and F). The FSL task results (Fig. 9, D and H) were obtained from a combined dataset comprising the modified FSL task (experiment 3) and the small-set FSL task (experiment 4) (see fig. S12, C, D, G, and H for individual results). The data from the original FSL task in experiment 2 were excluded because it lacked the conventional fixation period necessary to calculate baseline firing rates (see Fig. 4C, upper row).

In prior neuroimaging studies with awake monkeys (*43*, *46*), task-positive and task-negative brain regions were identified by contrasting activity between fixation (baseline) and active task periods, and we used this method as well. We used a baseline window of −600 to 0 ms before the end of fixation. As shown in Fig. 9, this choice excluded neural activity triggered by onset of the FP and the subsequent saccade, thereby preventing both overestimation and underestimation of the baseline level.

In Fig. 9 (A to D), we computed within-subject SEMs (inner error bars) (*63*, *64*). For each neuron *i* and time bin *j*, we first calculated the raw mean spike rate *y_ij_* and then normalized *y_ij_* to correct for neuron-specific firing rate differences using the formula

$$w_{\mathrm{ij}}=y_{\mathrm{ij}}-\left({\bar{y}}_{i.}-{\bar{y}}_{..}\right)$$

where $w_{\mathrm{ij}}$ represents the normalized spike rate, ${\bar{y}}_{i.}$ is the mean spike rate for neuron *i* (averaged across time bins), and ${\bar{y}}_{..}$ is the grand mean of all firing rates. Next, using $w_{\mathrm{ij}}$, the SEM ($\sigma /\sqrt{N}$) was calculated for each time bin, which was then multiplied by a correction factor ($\sqrt{M/\left(M-1\right)}$), where *N* is the number of neurons and *M* is the number of time bins. Because our primary interest was in pairwise comparisons of population firing rates between the baseline period and each time bin, we calculated a value ${\bar{y}}_{i.}$ using two values: firing rates in the baseline period and the *j*th bin (i.e., *M* = 2). Note that $w_{\mathrm{ij}}$ was used only for error-bar visualization, while the original raw firing rate values were used for all other plots and statistical tests in the figure.

In Fig. 9 (A to D), we tested whether population firing rates in pos-PFC and FPC deviated from the fixation baseline over time using cluster-based paired permutation tests on time-resolved firing rates computed in nonoverlapping 50-ms bins. For each bin, we computed a paired *t*-statistic across neurons on the within-neuron difference between the bin firing rate and the baseline firing rate. Clusters were defined as contiguous bins exceeding a two-tailed, uncorrected cluster-forming threshold of α = 0.05. The cluster statistic was cluster mass, defined as the sum of |*t*| across bins within a cluster. The null distribution of the maximum cluster mass was generated by sign-flipping the paired differences within each neuron across 5000 permutations. For each observed cluster, the cluster mass was compared against the null distribution of the maximum cluster mass across permutations to obtain a cluster-level *P* value.

In Fig. 9 (E to H), to determine whether the activity of each neuron showed significant task-related activation or deactivation relative to baseline, we compared each neuron’s overall firing rate during six task periods against baseline (for each period, paired-permutation test, *P* < 0.05). The six task periods were as follows: (i) cue (0.1 to 0.4 s from cue onset), (ii) delay (0.4 to 1.0 s from cue onset), (iii) late delay (−0.3 to 0 s from go signal), (iv) pre-acq (−0.3 to 0 s from saccadic target acquisition), (v) post-acq (0 to 0.3 s from saccadic target acquisition), and (vi) post-reward period (0 to 0.3 s from reward). In Fig. 9 (B and F) (attention task), because the task used a lever-release response, the pre-acq and post-acq periods were named the pre-lever (Pr-L) and post-lever (Po-L) periods, respectively.

### Analysis of neural selectivity for task variables

To quantify the strength of neural selectivity for a given task variable, we used the PEV. For our main analyses (Figs. 3 to 8), we computed the partial ω^2^ value for each factor in an *n*-way ANOVA model. The calculation was performed using the formula

$$\omega_{p}^{2}=\frac{{\text{SS}}_{\text{effect}}-{\text{df}}_{\text{effect}}\times \text{MSE}}{{\text{SS}}_{\text{effect}}+\left(N-{\text{df}}_{\text{effect}}\right)\times \text{MSE}}$$

where SS_effect_ and df_effect_ are the type II sum of square and the degree of freedom (between groups), respectively, for the factor of interest. MSE is the mean square error within groups, and *N* is the total number of trials. The partial ω^2^ indicates how much variance in a neuron’s trial-by-trial firing rate is explained by a given factor, while accounting for the influence of other factors in the ANOVA model. For the analysis in figs. S3A, S10, and 11, with a single analytical variable, we computed the standard omega-squared (ω^2^) for a one-way ANOVA model using the following formula

$$\omega^{2}=\frac{{\text{SS}}_{\text{effect}}-{\text{df}}_{\text{effect}}\times \text{MSE}}{{\text{SS}}_{\text{total}}+\text{MSE}}$$

where SS_total_ is the total sum of squares for the entire dataset. For the time-series analysis, PEV was computed using 200-ms time windows slid in 20-ms increments across the entire trial. In each time window, the observed population-averaged PEV values were compared against a null distribution created by randomly shuffling condition labels 10,000 times, thereby simulating the distribution of PEV values expected by chance (one-sample permutation test). The resultant permutation *P* values were then corrected for multiple comparisons across time windows by controlling FDR under a BH procedure. The significance level was set at *P* < 0.05. Depending on the nature of the comparison (within-subject versus between-subject), we additionally used paired or two-sample permutation tests as appropriate.

## Supplementary Materials

### The PDF file includes:

Supplementary Notes S1 to S4 Figs. S1 to S13 Table S1
