# Supplementary material for: Reversed functional gradient in primate prefrontal cortex: Posterior dominance and frontopolar task-related deactivation
Source: Sci Adv. 2026 Jul 17;12(29):eaea1094. doi: 10.1126/sciadv.aea1094 (PMC13378552; doi:10.1126/sciadv.aea1094)
Supplement: Supplementary file 1 — Supplementary Notes S1 to S4 Figs. S1 to S13 Table S1 [file sciadv.aea1094_sm.pdf]

Science Advances
AAAS

Supplementary Materials for

Reversed functional gradient in primate prefrontal cortex: Posterior dominance and frontopolar task-related deactivation

Kei Watanabe *et al.*

Corresponding author: Kei Watanabe, watanabe.kei.fbs@osaka-u.ac.jp

Sci. Adv. **12**, eaea1094 (2026)
DOI: 10.1126/sciadv.aea1094

The PDF file includes:

Supplementary Notes S1 to S4
Figs. S1 to S13
Table S1

Note S1. Supplementary text for discussion section

In Experiments 1 and 2, dual-task-specific modulations of neuronal activity emerged almost exclusively in the pos-PFC (**Fig. 3, E to I** and **Fig. 4I**). A sole prior study examining the role of the monkey FPC in cognitive orchestration (*8*) inserted a secondary task (face-detection or free-reward consumption task) into ITIs of the Wisconsin card sorting task (WCST). Following secondary-task insertion, WCST performance declined relative to the no-insertion condition in both FPC-lesioned and intact control groups; counterintuitively, however, the magnitude of this post-insertion decrement was smaller in FPC-lesioned monkeys than in controls, resulting in higher post-insertion WCST performance in the lesion group.

The authors proposed that the FPC is critical for disengaging from the ongoing task (WCST) and reallocating cognitive resources to other potential goals, such as the inserted secondary task. Accordingly, during secondary-task insertion, FPC-lesioned monkeys were less distracted by extra-WCST events and remained more engaged in the WCST, resulting in better post-insertion performance. However, several points warrant consideration before accepting this account. First, if the FPC truly reallocates resources to the inserted secondary task, FPC lesions should impair allocation of cognitive resources to the secondary task, thereby impairing secondary-task performance. Yet, no quantitative comparisons were provided; it was only noted that both lesioned and control monkeys performed face detection at over 90% accuracy and consumed all the reward, implying little to no resource-reallocation deficit. Second, the resource allocation account would also predict that the FPC is needed to shift resources back to the WCST after the completion of inserted secondary task. On this view, FPC-lesioned monkeys should have been impaired when returning to the WCST, making their superior post-insertion WCST performance difficult to reconcile with the account. The absence of any such "return" deficit, which would imply that resource reallocation is unidirectional (from the WCST to the secondary task only), remains unexplained.

In our study, if FPC activity was directing cognitive orchestration, it should at least distinguish between single- and dual-task conditions. However, in Experiments 1 and 2, it did not. Instead, neural activity reflecting dual-task-specific processes and adaptive resource allocation emerged predominantly at AP levels corresponding to the posterior and middle thirds of the principal sulcus, and was almost absent in the anterior third and beyond (**Fig. 3, G and I** and **Fig. 4I**). Along with the lack of significant interaction effects in the FPC throughout Experiments 1–5, this suggests that the monkey FPC contributes minimally to multiple-goal management and information integration during cognitive orchestration. Rather, these processes are likely to be supported primarily by the posterior-to-mid LPFC.

In novel learning (Experiment 3), 8% of FPC neurons responded preferentially to the appearance of novel objects (negative-slope neurons, **Fig. 5, D to F**), consistent with Nougaret et al. (*13*), who reported a comparable proportion of such neurons in their full FPC sample. At first glance, the FPC appears to be crucial for novel learning. However, we have demonstrated that in the pos-PFC, a significantly higher proportion of negative-slope neurons responded to novel objects at an earlier timing than in the FPC (Epoch A, **Fig. 5E**). Furthermore, while reward-prediction-error (RPE)-related neurons which likely support novel S–R learning emerged in the FPC, their prevalence was significantly greater in the pos-PFC than FPC (**Fig. 6B**). In Experiment 4, when the object set-size was drastically reduced from that in Experiment 3, the pos-PFC adaptively changed activity patterns and remained engaged in learning, whereas the FPC showed minimal involvement (**Fig. 7, D and G**). These findings suggest that the FPC plays a much more minor role in learning processes than the pos-PFC. Nevertheless, given that the ventral corticostriatal circuit, including OFC, is

typically the primary driver of value-based object learning (*28*,*29*), even the posterior LPFC's role seems to remain relatively modest.

In the spatial and object CS tasks (Experiment 5), our findings challenge a prior proposal (*11*) that the FPC is crucial for monitoring and evaluating self-generated decisions to guide future choices in dynamic environments. In our novel object CS task emphasizing object information, FPC activity around feedback, previously linked to decision monitoring and outcome evaluation, only minimally encoded chosen objects or their correctness (**Fig. 8, H to J**). Instead, it predominantly represented task-irrelevant chosen-location information. In contrast, pos-PFC activity robustly encoded both monitoring and evaluation processes in both spatial and object CS tasks, while also exhibiting flexible resource allocation by shifting the weighting of representations across modalities (space and object) in response to changing task demands (**Fig. 8K**). Furthermore, contrary to earlier proposals (*11*), an action need not be 'self-generated' to be represented in the FPC: comparable chosen-location-selective peri-reward activity appeared in the externally instructed MGS task and the spatial CS task under the delayed reward condition (**Fig. 8M**, and **figs. S10** and **S11**). A prior study on object coding in monkey FPC found minimal object selectivity during an object memory task (*12*). Our findings confirm and extend this, showing that FPC neurons rarely exhibit object selectivity, even during the initial phase of learning (Experiment 4) and self-generated decision-making (Experiment 5), contexts where the monkey FPC was previously considered crucial.

Note S2. Supplementary text for fig. S3

One potential concern in our analysis of Experiment 1 (**Fig. 3, E, H and I**) is that in most recording sessions (60/75), the single-task and dual-task MGS conditions were conducted in relatively long, separate blocks. This setup may have introduced small but gradual changes in firing rate (drift) between the two conditions, potentially affecting the results, particularly those related to the factor task that compared overall firing-rate differences between the single-task and dual-task MGS. To address this issue, we repeated the analysis using only neurons recorded during the frequent task-switching sessions (n = 15 sessions) in which single-task and dual-task blocks alternated frequently (8 ± 1.2 times per session; every 40–50 correct trials). The results (**fig. S3**) closely matched those in **Fig. 3**. The population PEV time courses (**fig. S3A)** were comparable to those in **Fig. 3E**, and the time course of percentages of dual-task preferring neurons (**fig. S3, B and D)** were also comparable to those in **Fig. 3, F and H**. Notably, the percentages of neurons exhibiting the dual>single firing-rate pattern again showed a significant anterior decline (magenta plot, **fig. S3E**), as in **Fig. 3I**. The percentages of neurons exhibiting the dual>single location-selectivity pattern also showed anterior decline, though not significant (magenta plot, **fig. S3C**). Additionally, as in **Fig. 3H**, pos-PFC activity exhibited heightened readiness during the initial fixation period in the dual-task MGS compared to the single-task MGS (arrows in **fig. S3D**), which likely reflected the expectation of higher mental workloads in dual-task MGS. These findings indicate that potential neuronal drift had negligible impact on our results.

Note S3. Supplementary text for fig. S7

One concern in the analysis of **Fig. 5, C to F** (Experiment 3) is the inclusion of data from both fixation (Experiment 3) and non-fixation (Experiment 2) conditions before and after array onset in the FSL task. This could confound the neuronal results, as eye movement frequency after array onset may vary depending on the trial's ordinal position following the problem change. Notably, this issue also applies to the previous FPC study, where fixation was not required at all throughout the object-in-place task (*13*).

To address this concern, we repeated the analyses using only Experiment 3 data (**Fig. 5A**, modified FSL task). The results (**fig. S7**) were essentially unchanged, ruling out eye movement frequency as a confounding factor. Specifically, for the factor ordinal trial position, the significant PEV elevation in the pos-PFC again preceded that in the FPC by 180 ms (vertical arrow, middle panel, **fig. S7A**). The percentages of negative- and positive-slope neurons during these slightly shifted Epochs A and B (100–280 ms and 280–760 ms from array onset, respectively) were comparable to those in **Fig. 5E** (**fig. S7B**). Other findings (**fig. S7C**) also aligned with **Fig. 5F**. These results indicated that our main findings related to novel learning (**Fig. 5, C to F**) were not affected by factors concerning eye movement.

Note S4. Supplementary text for fig. S12

The reason why FPC activity did not show deactivation in the FSL task (**Fig. 9, D and H**) is likely because the cue (object-array) period in this task involved more intense visual stimulation compared to the remaining three tasks (**Fig. 9, A to C**). In the modified FSL task (**Fig. 5A**), three objects (3.8° on a side) were presented simultaneously within six possible presentation locations (3 locations × 2 configurations), covering a large portion of the visual field. In the small-set FSL task (Experiment 4, **Fig. 7B**), two objects with slightly smaller size (3.2° on a side) than in the modified FSL task (**Fig. 5A**) were presented within three possible locations. These intense visual stimuli elicited a stronger visual response (arrow in **Fig. 9D**) than those elicited in the other three tasks (**Fig. 9, A to C**), and therefore likely overrode subsequent inhibitory modulation.

Separate analyses confirmed that the FPC visual response was significantly stronger during the modified FSL task (3 objects in 6 possible locations) compared to the small-set FSL task (2 slightly smaller objects in 3 possible locations) (grey curves, **fig. S12, C and D**). In the modified FSL task, this strong visual response gradually decayed but persisted until the pre-acq period (**fig. S12C)**. In contrast, in the small-set FSL task, weaker visual stimulation elicited only a modest visual response (**fig. S12D**), while the overall firing rate remained near baseline throughout the trial. These findings suggest that the strong object-array-evoked visual response in the FSL task acted as a confounding factor in this baseline-referenced analysis, masking any subsequent inhibitory modulation and thereby obscuring task-related deactivation.

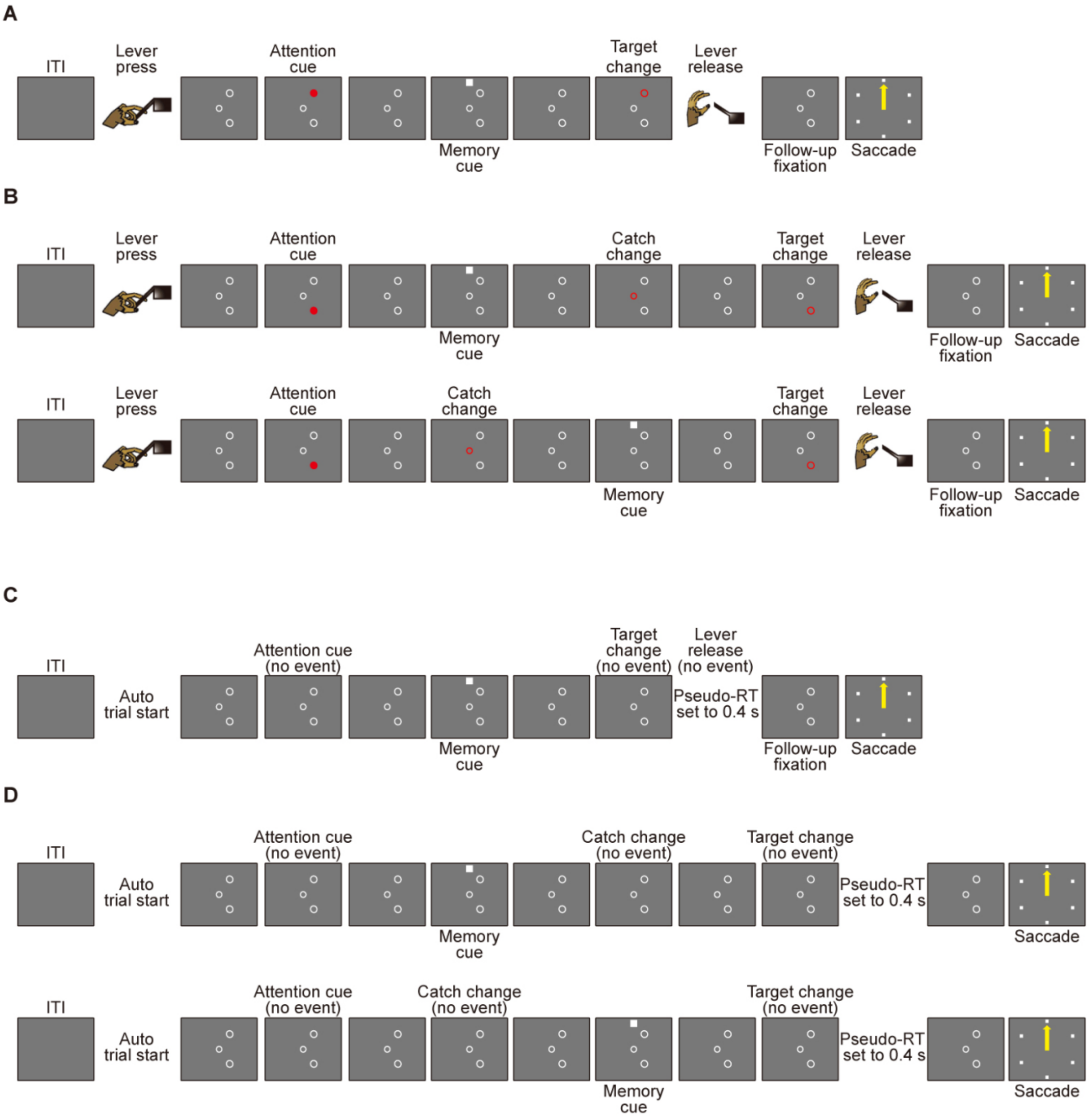

Figure S1 Example dual-task MGS and single-task MGS trial sequences.
(A) MGS task (memory cue) inserted into a short trial of the attention task. This type of dual-task MGS comprised half of the entire dual-task MGS trials (33.3% of all attention-task trials). (**B**) MGS task (memory cue) inserted into a long trial of the attention task. This type of dual-task MGS comprised the remaining half of all dual-task MGS trials (33.3% of all attention-task trials). Thus, together, these two types of dual-task MGS trials accounted for 66.7% of all attention-task trials. (**C**) Trial sequence of the single-task MGS performed in a separate block of trials. This type of single-task MGS trial was matched in the time course and trial length to the short attention-task trial (and thus to the dual-task MGS trial shown in panel **A**). This type comprised 50% of all single-task MGS trials. (**D**) Trial sequence of the single-task MGS trial, matched in time course and trial length to the long attention-task trial (and thus to the dual-task MGS trial shown in panel B). This type comprised the remaining 50% of all single-task MGS trials. In these single-task MGS trials, all attention-task events were scheduled but implemented as 'empty events' with no physical changes in stimuli. Trials were automatically initiated by the appearance of the fixation ring after an intertrial interval (ITI).

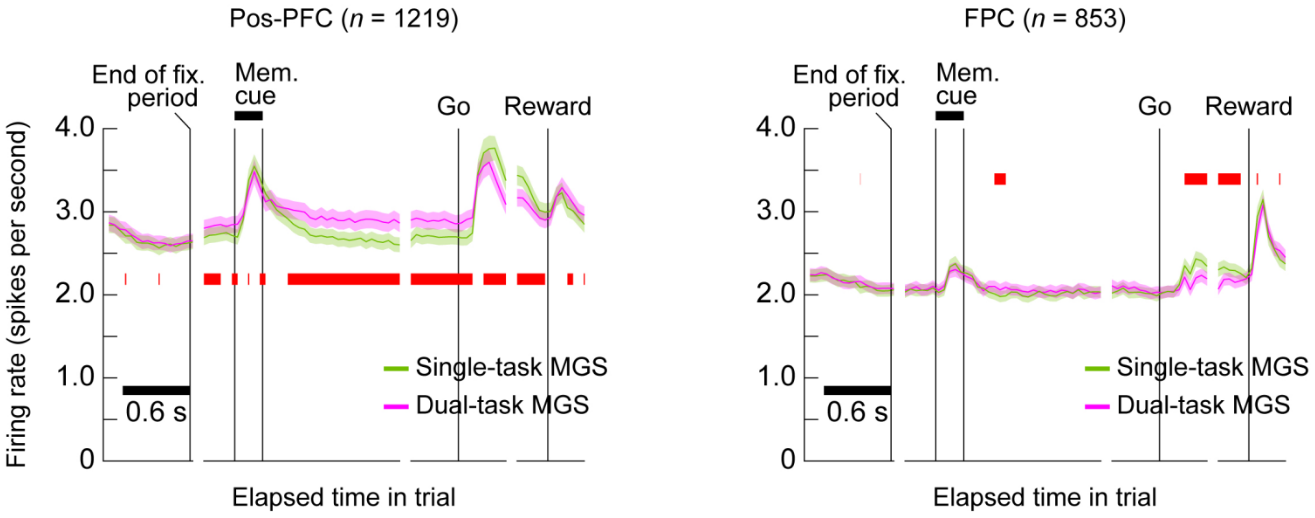

Figure S2 Comparison of overall firing rates between single-task MGS and dual-task MGS.
Left panel: Pos-PFC. Population-averaged overall firing rate during the single-task (green) and dual-task MGS (magenta) averaged across all cue locations, aligned as in **Fig. 3E** (to the end of fixation period, memory-cue onset, go signal, and reward delivery). Shading indicates SEM. Red horizontal bars mark time bins with significant difference between the two MGS conditions (two-sample permutation test, $p < 0.05$; FDR-corrected). Right panel: Same as in the left panel, but for FPC. Bin width = 50 ms (non-overlapping). Analyses excluded neural responses evoked by attention-task events (**Materials and Methods**). Fix., fixation; Mem., memory.

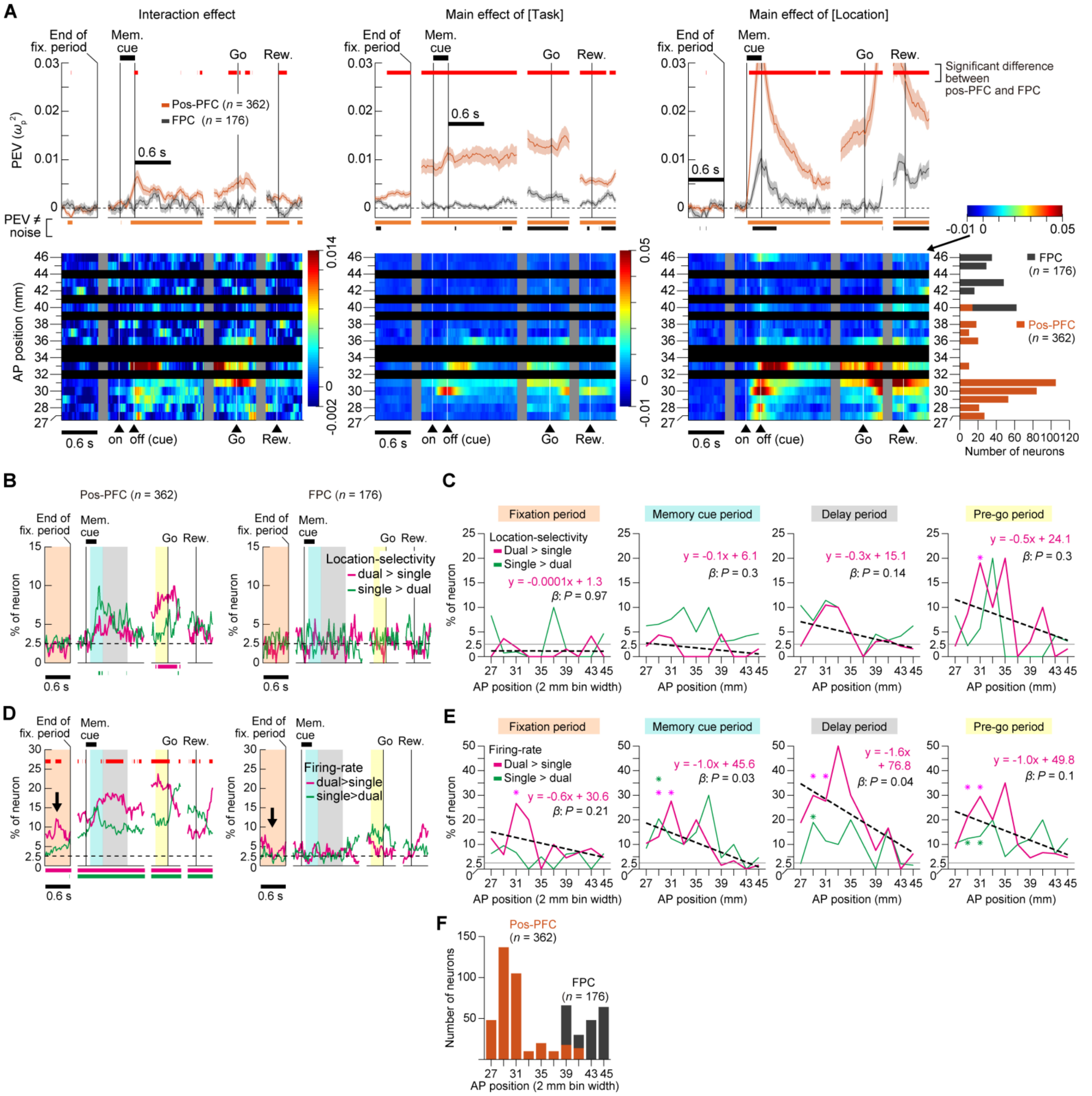

Figure S3 Reanalysis of Experiment 1 restricted to the frequent task switching sessions.
(A) Upper row: Time course of population-averaged PEV for interaction (left), and main effects of task (middle) and location (right). For the pos-PFC, data from the dorsal and ventral pos-PFC are combined. Bottom row: Time course of population-averaged PEV in each 1-mm segment of recording locations along the AP axis. Horizontal black strips in the plot area indicate locations where no neurons were recorded. Other conventions as in **Fig. 3E**. (**B**) Time course of the percentages of neurons exhibiting the dual>single (magenta) and single>dual (green) location-selectivity patterns. Conventions as in **Fig. 3F**. (**C**) Changes in the percentages of the dual>single (magenta) and single>dual (green) location-selectivity patterns as a function of AP position, during the fixation (orange), memory cue (cyan), delay (gray) and pre-go (yellow) periods. Due to the limited number of neurons, percentages were computed using 2-mm bins rather than 1-mm bins. (**D** and **E**) Same as in **B** and **C**, respectively, but for the dual>single (magenta) and single>dual (green) firing-rate patterns. (**F**) Number of recorded neurons in each 2-mm segment along the AP axis.

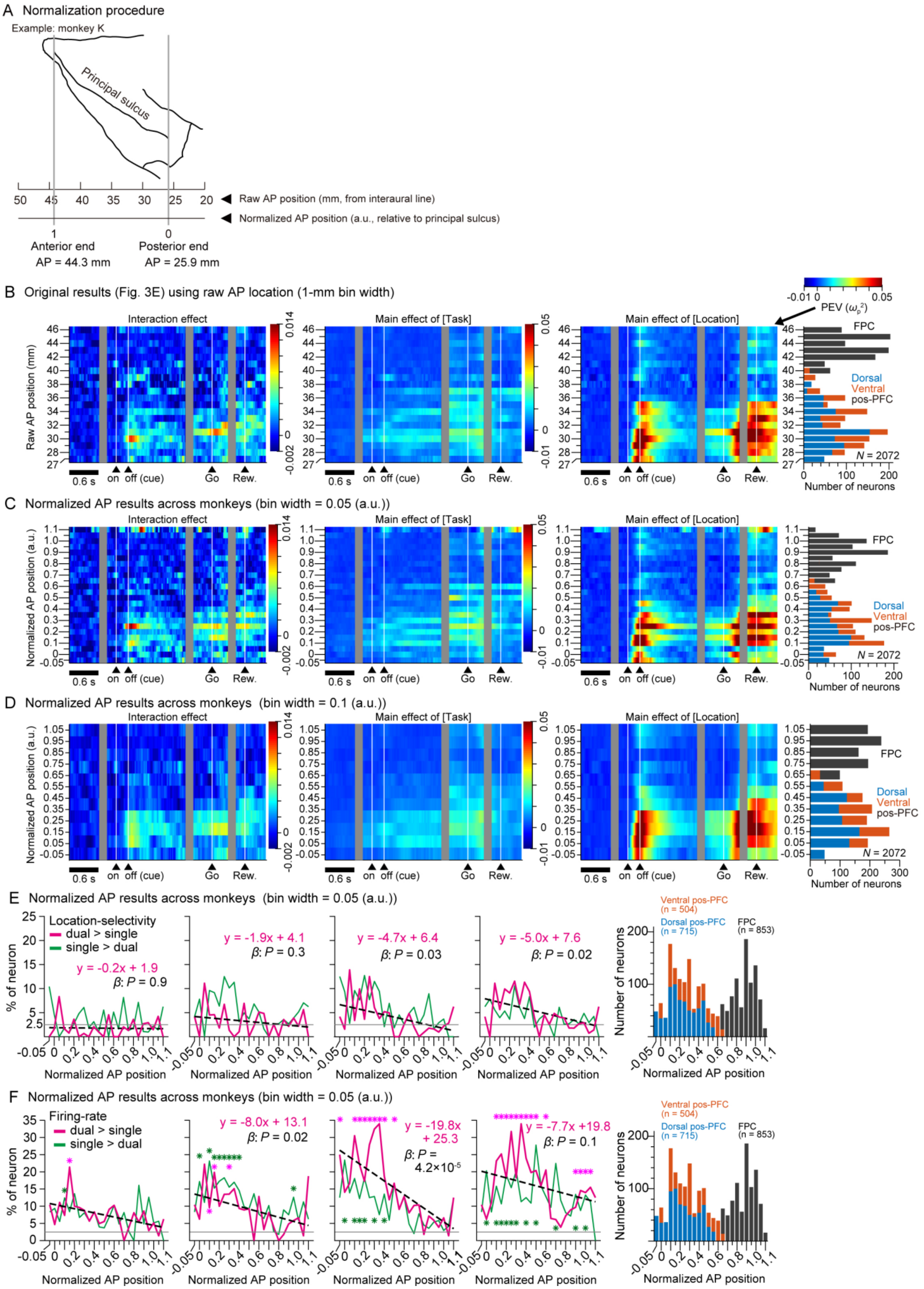

Figure S4 Reanalysis of Experiment 1 using normalized AP coordinates. (**A**) Illustration of the normalization method. For each recording site, we subtracted the AP coordinate of the posterior end of the

principal sulcus of the corresponding monkey and divided the value by the distance between the posterior and anterior ends of the principal sulcus. (**B**) Original results shown in **Fig. 3E** using raw AP coordinates in millimeter, reproduced here for reference. (**C**) Same data as in **B** replotted using normalized AP coordinates across monkeys K and T (bin width = 0.05, arbitrary units). (**D**) Same as in **C**, but using a bin width of 0.1 (arbitrary units). Note the close resemblance of the results obtained using normalized AP coordinates to those in panel **B**. (**E**) The results in **Fig. 3G** (using raw AP value) replotted using normalized AP coordinates (bin width = 0.05, arbitrary units). (**F**) The results in **Fig. 3I** (using raw AP value) replotted using normalized AP coordinates (bin width = 0.05, arbitrary units).

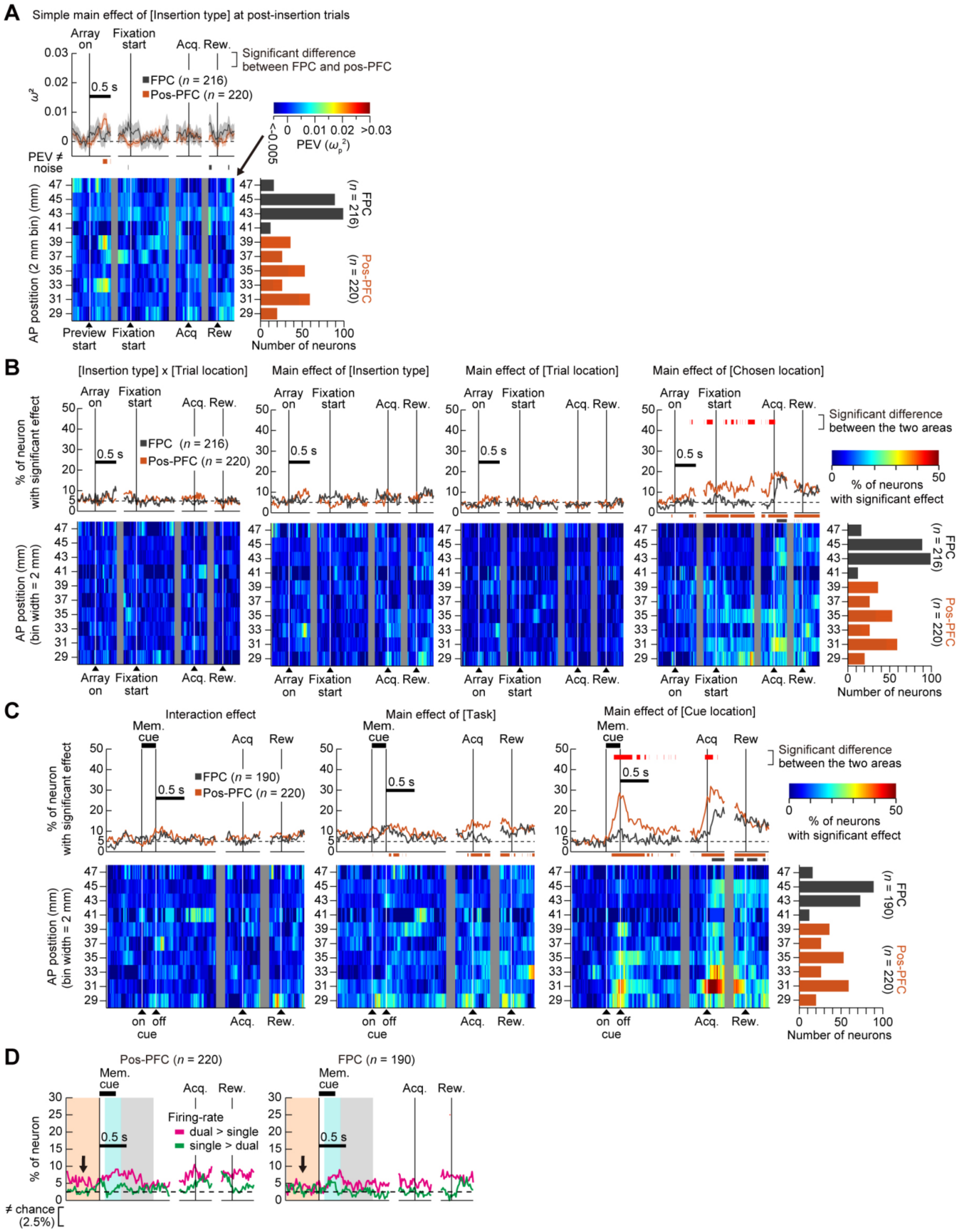

Figure S5 Experiment 2: Additional analyses for neural activity during the serial multimodal dual task. (**A**) Time course of population PEV for the simple main effect of the factor insertion type, using only post-insertion trials. Conventions as in **Fig. 4H**. (**B**) Time course of the percentages of significant neurons for the four

critical ANOVA terms in the FSL task, aligned at the start of the preview period (array on) and wait period (fixation start), and the timing of target acquisition (acq.) and reward (rew.). Top row: Red horizontal bars mark time periods with significant differences in the percentages between FPC (gray curve) and pos-PFC (brown curve) (Fisher's exact test, FDR-corrected $p < 0.05$). Lower colored horizontal bars mark time periods in which the percentage was significantly different from chance (5%) in each recording area (Fisher's exact test, FDR-corrected $p < 0.05$). Bottom row: Time course of the percentage of significant neurons in each 2-mm segment of recording locations. The rightmost panel indicates the number of recorded neurons. (**C**) Same as in **B**, but for the comparison between single-task and dual-task MGS. (**D**) MGS task: Time course of the percentages of neurons exhibiting the dual>single firing-rate pattern (magenta) and single>dual firing-rate pattern (green) in the pos-PFC (left) and FPC (right, shown for reference). In the pos-PFC, the percentages for the dual>single firing-rate pattern consistently outnumbered those for the single>dual firing-rate pattern throughout the trial, though not significantly. This result resembled the pattern observed in Experiment 1. Notably, also similar to Experiment 1, during the fixation period, the pos-PFC showed an elevated (though not significant) percentage of the dual>single firing-rate pattern compared to the single>dual firing-rate pattern (arrows), suggesting that the pos-PFC activity was primed from the foreperiod for the anticipated higher cognitive demand of dual-task conditions.

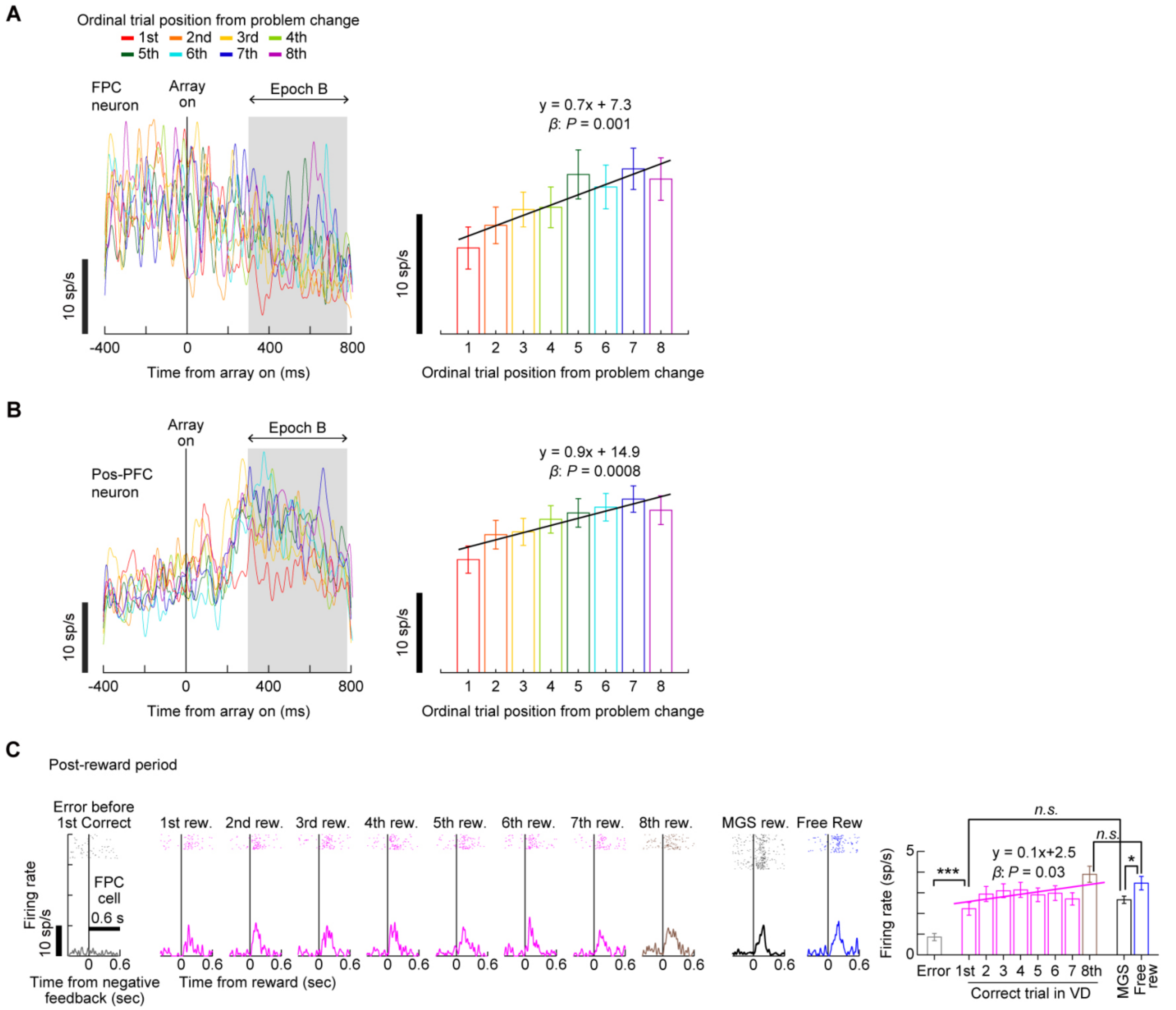

Figure S6 Representative positive-slope neurons at array onset and post-reward in Experiment 3. (**A** and **B**) Activity of representative positive-slope neurons aligned at array onset in the FPC (**A**) and pos-PFC (**B**) during trials 1 to 8 after problem change. The right panel shows the mean firing rate in Epoch B (shaded area) with OLS regression results. (**C**) Post-reward activity of a representative positive-slope neuron in the FPC across 1st to 8th correct trials after problem change, shown alongside activity in error trials (leftmost panel) and in MGS reward and free reward. The rightmost panel indicates the mean firing rate for each trial type in Epoch C (0 – 0.6 s from reward) with OLS regression results.

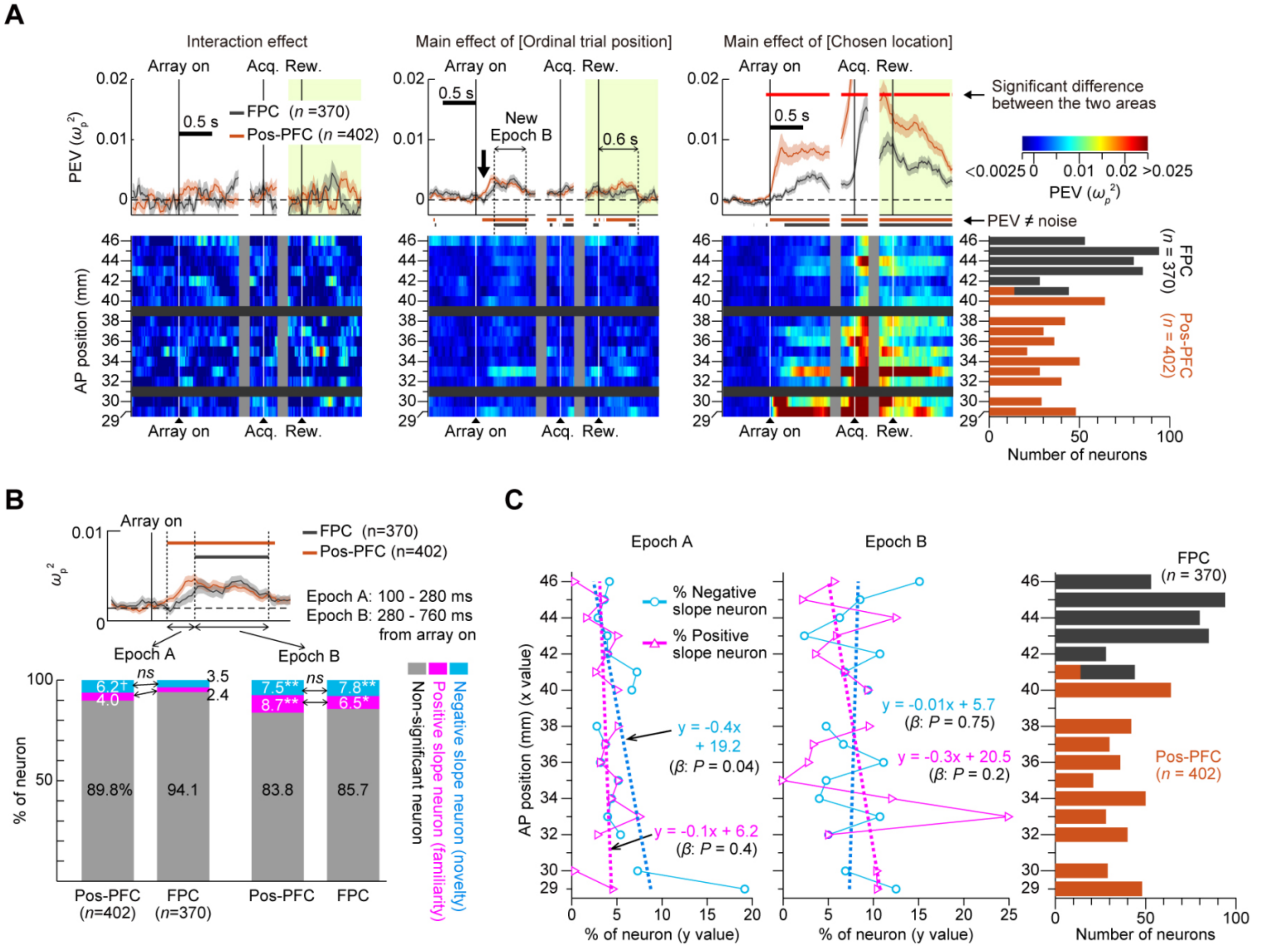

Figure S7 Neural activity across the anteroposterior LPFC in the modified FSL task (only data from Experiment 3 with fixation requirement before and after array onset).

(**A**) Time course of population-averaged PEV. Conventions as in **Fig. 5C**. (**B**) Comparison of the percentage of negative- (cyan) and positive-slope (magenta) neurons between the pos-PFC and FPC in Epochs A and B. Conventions as in **Fig. 5E**. The inset PEV plot enlarges the region near 'array on' in **A** (second panel from left). (**C**) Percentage of negative- (cyan) and positive-slope(magenta) neurons across 1-mm segments along the AP axis for Epochs A (left panel) and B (middle panel). No FPC subregion exceeded the 2.5% chance level for either negative- or positive-slope neurons in either epoch (for both epochs: $p > 0.28$, p-values corrected for 16 comparisons in each epoch). Conventions as in **Fig. 5F**. Rightmost panel shows the number of neurons recorded per 1-mm segment.

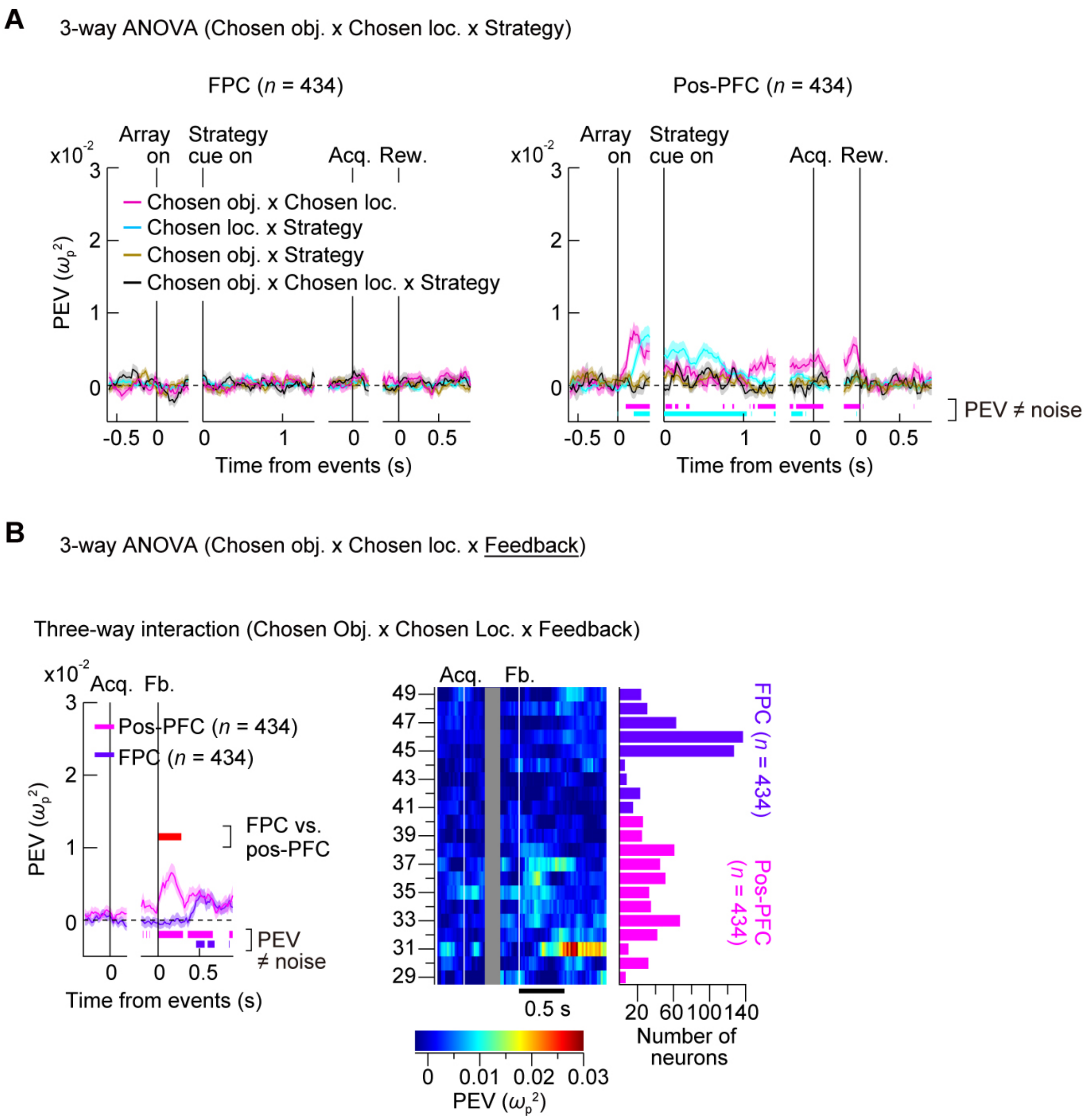

Figure S8 Interaction effects observed during the object CS task across the anteroposterior LPFC. (**A**) Time course of population PEV for the three first-order interaction terms and one second-order (three-way) interaction term from the 3-way ANOVA used in **Fig. 8H**, shown separately for the FPC (left) and pos-PFC (right). Lower colored horizontal bars indicate periods of significant PEV for each term with a corresponding color. Other conventions as in **Fig. 8H**. Obj., object; loc., location. (**B**) Left panel: Comparison of PEV for the second-order (three-way) interaction term ([chosen object × chosen location × feedback]) between FPC (purple) and pos-PFC (magenta). Conventions as in **Fig. 8I**. Right panel: PEV for this interaction term at each 1-mm segment along the AP axis. Conventions as in **Fig. 8J.**

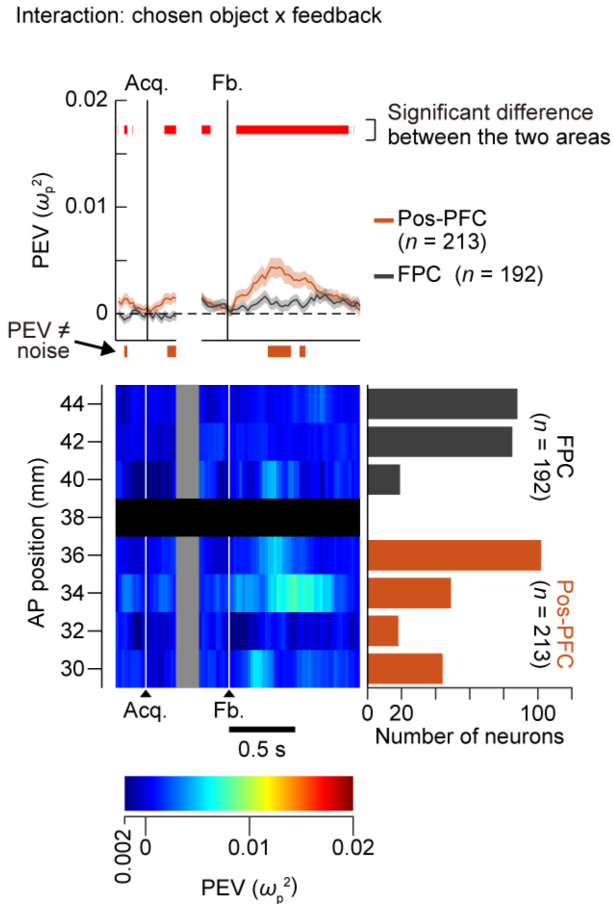

Figure S9 Differential encoding of decision-outcome evaluation between FPC and pos-PFC in the small-set FSL **task (Experiment 4).**
Top panel: Time course of PEV for the critical interaction term (chosen object × feedback) in the 3-way ANOVA with factors: chosen object, feedback, and ordinal trial position. Conventions as in **Fig. 7D**. Bottom panel: Time course of PEV for this interaction term at each position (2-mm bin).

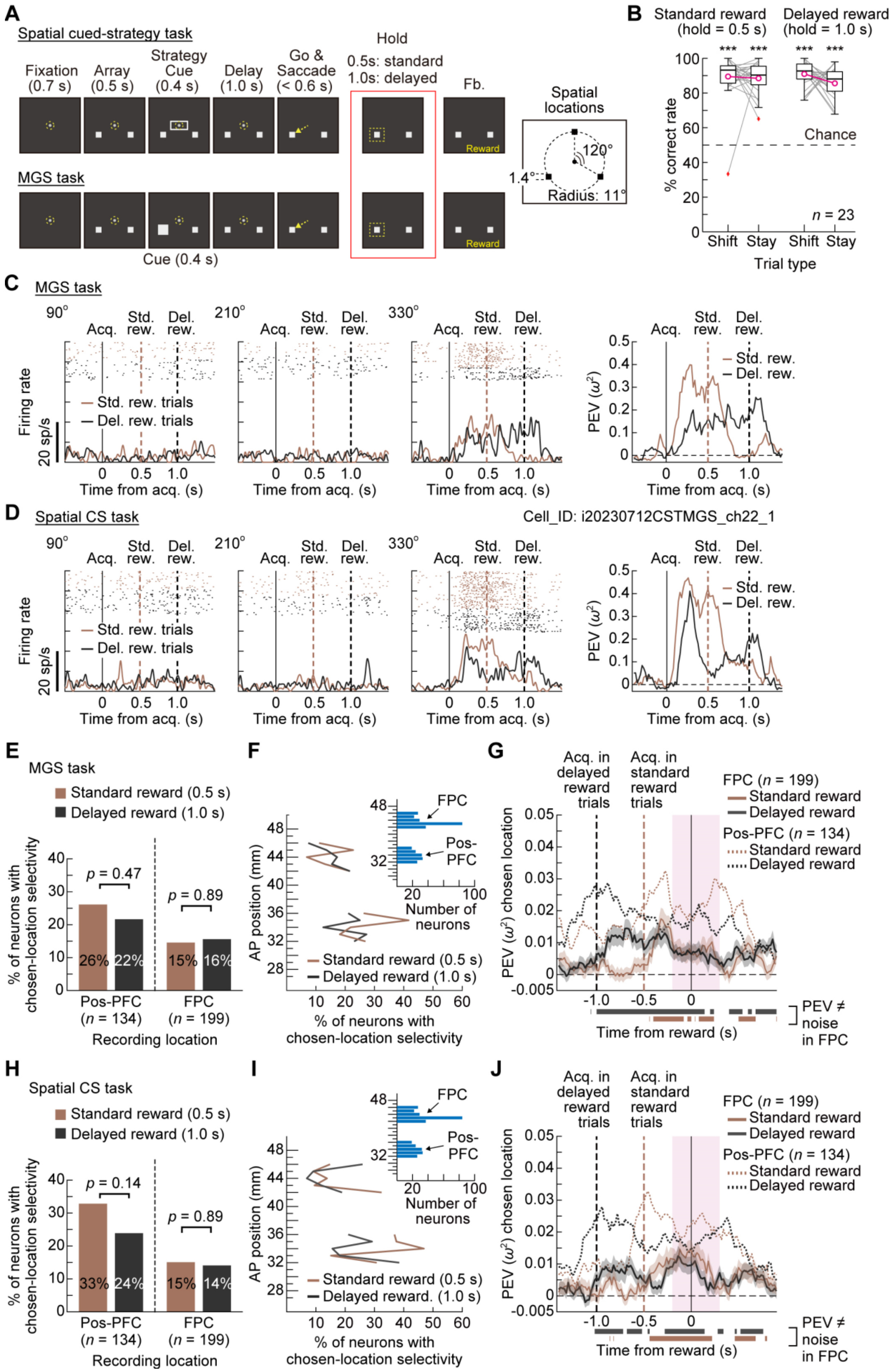

Figure S10 FPC neurons showed sustained chosen-location selectivity until delayed reward even in the MGS task (Experiment 5).

(**A**) Standard (0.5-s hold period) and delayed (1.0-s hold) reward (feedback) conditions in the spatial CS (top row) and MGS tasks (bottom row). In this version of the MGS task, two placeholders were presented at trial start to match the display sequence in the spatial CS task. During the cue period, a memory cue (twice the placeholder size) appeared randomly on one of the two placeholders. Trials were grouped into a problem comprising 12-16 correct trials. Two placeholder locations stayed the same within each problem. (**B**) Behavioral performance of monkey Um in the spatial CS task under the standard (left) and delayed (right) feedback conditions. Performance in all four task conditions was significantly above chance (Wilcoxon signed-rank test; $p < 0.0002$, corrected). (**C**) MGS task: Raster-histograms in each cue location (three left panels) and the PEV for chosen location (cue location) aligned at saccadic target acquisition (acq.) for a representative FPC neuron. Data for the standard and delayed reward (feedback) conditions are shown in brown and black, respectively. Brown and black vertical dashed lines indicate the onset of the standard (std.) and delayed reward (del. rew.), respectively. (**D**) Same neuron as in **C**, but for the spatial CS task. (**E**) MGS task: Percentage of neurons with significant chosen-location selectivity in the peri-reward period (-0.2 to 0.3 s from reward; pink shaded area in panel **G**) for the standard and delayed reward conditions in the pos-PFC (two left bars) and FPC (two right bars). *P*-values are for Fisher's exact test (uncorrected). (**F**) MGS task: Percentage of neurons with significant chosen-location selectivity in the peri-reward period in each 1-mm segment along the AP axis, separately shown for the standard (brown) and delayed (black) reward conditions. No significant difference was found between the two conditions across all 10 segments (Fisher's exact test, FDR-corrected). Inset shows the number of neurons recorded. (**G**) MGS task: Time course of population-averaged PEV for chosen location, aligned at reward delivery, in the FPC (solid lines) and pos-PFC (dotted lines). For clarity, the SEM (shaded area) is shown only for the FPC results. Brown and black vertical dashed lines indicate timings of acq. for the standard and delayed reward conditions, respectively. Lower horizontal bars indicate time periods of significant PEV in the FPC for each condition (one-sample permutation test, FDR-corrected). The pink shaded area indicates the peri-reward period. (**H** to **J**) Same as in **E** to **G**, but for the spatial CS task. In panel **I**, across all 10 segments, no significant difference was observed between the two reward conditions.

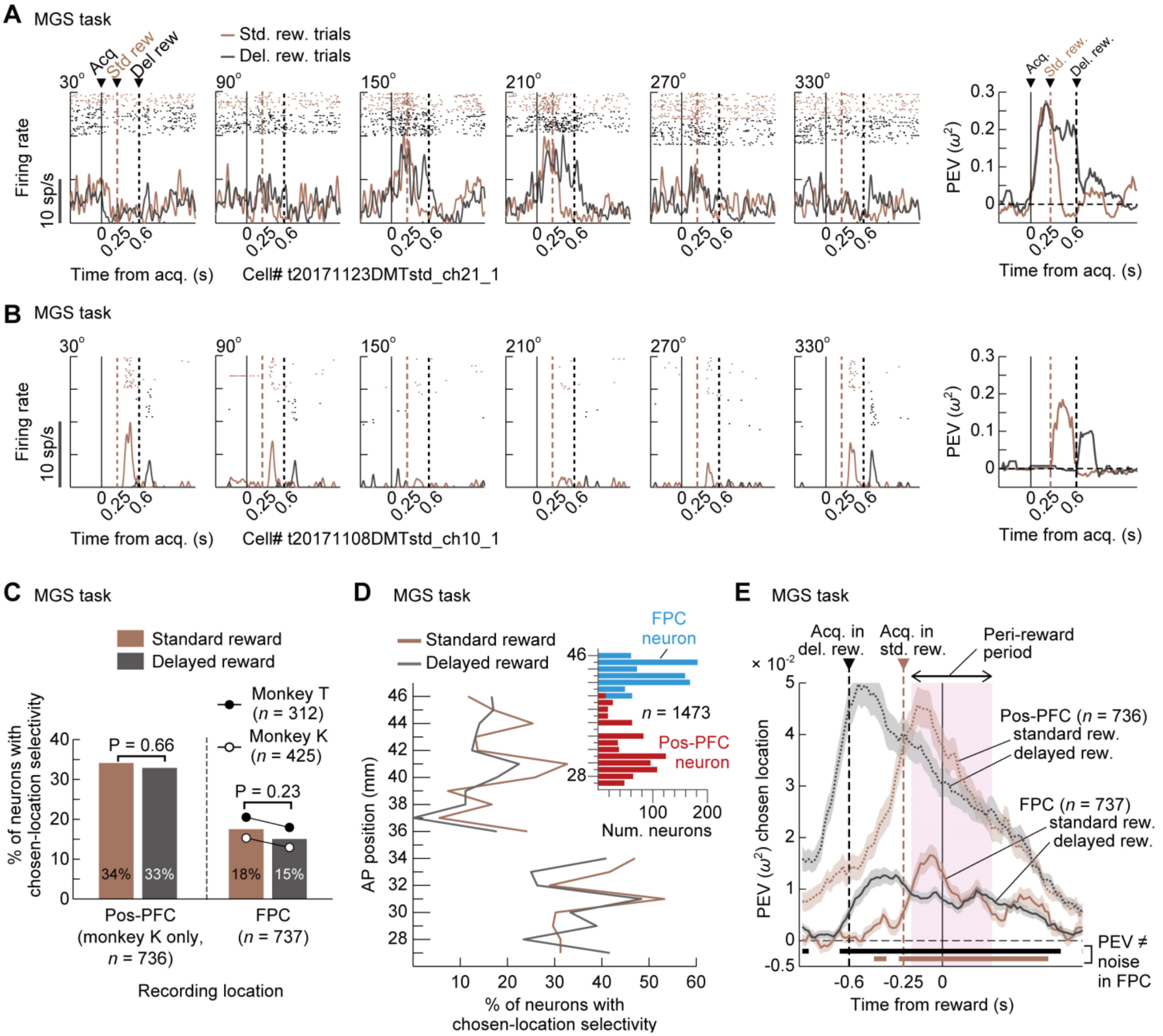

Figure S11 FPC neurons showed sustained chosen-location selectivity until delayed feedback even in the MGS task (Experiment 1).

(**A** and **B**) Raster-histograms and PEV for two representative FPC neurons in the standard (brown; 0.25-s hold period) and delayed reward (black; 0.6-s hold period) conditions in the MGS task, aligned at saccadic target acquisition (acq.). There were six possible cue locations in the MGS task (see **Fig. 2C**). Other conventions as in **fig. S10C**. (**C**) Percentage of neurons with significant chosen-location (cue location) selectivity in the peri-reward period (-0.2 to 0.3 s from reward; pink shaded area in **e**) for standard (brown) and delayed reward (black) conditions in the pos-PFC (two left bars) and FPC (two right bars). Conventions as in **fig. S10E**. (**D**) Percentage of neurons with significant chosen-location selectivity in the peri-reward period in each 1-mm segment along the AP axis. Across all 19 segments, no significant difference between the two reward conditions was observed (Fisher's exact test, FDR-corrected). Conventions as in **fig. S10F**. (**E**) Time course of population-averaged PEV (mean ± s.e.m.) for chosen location in the standard (brown) and delayed reward (black) conditions, aligned at reward delivery. Results are separately shown for the FPC (solid lines) and pos-PFC (dotted lines). Lower horizontal bars indicate time periods of significant PEV in the FPC for each reward condition. Other conventions as in **fig. S10G**. Again as in fig. S10, in the MGS task, despite the absence of self-generated decision-making, significant chosen-location selectivity in the FPC (and in pos-PFC) persisted even until the delayed reward. This suggests that simply tracking how long chosen-location selectivity remains significant after target acquisition is unrelated to assessing the FPC's role in monitoring self-generated decisions.

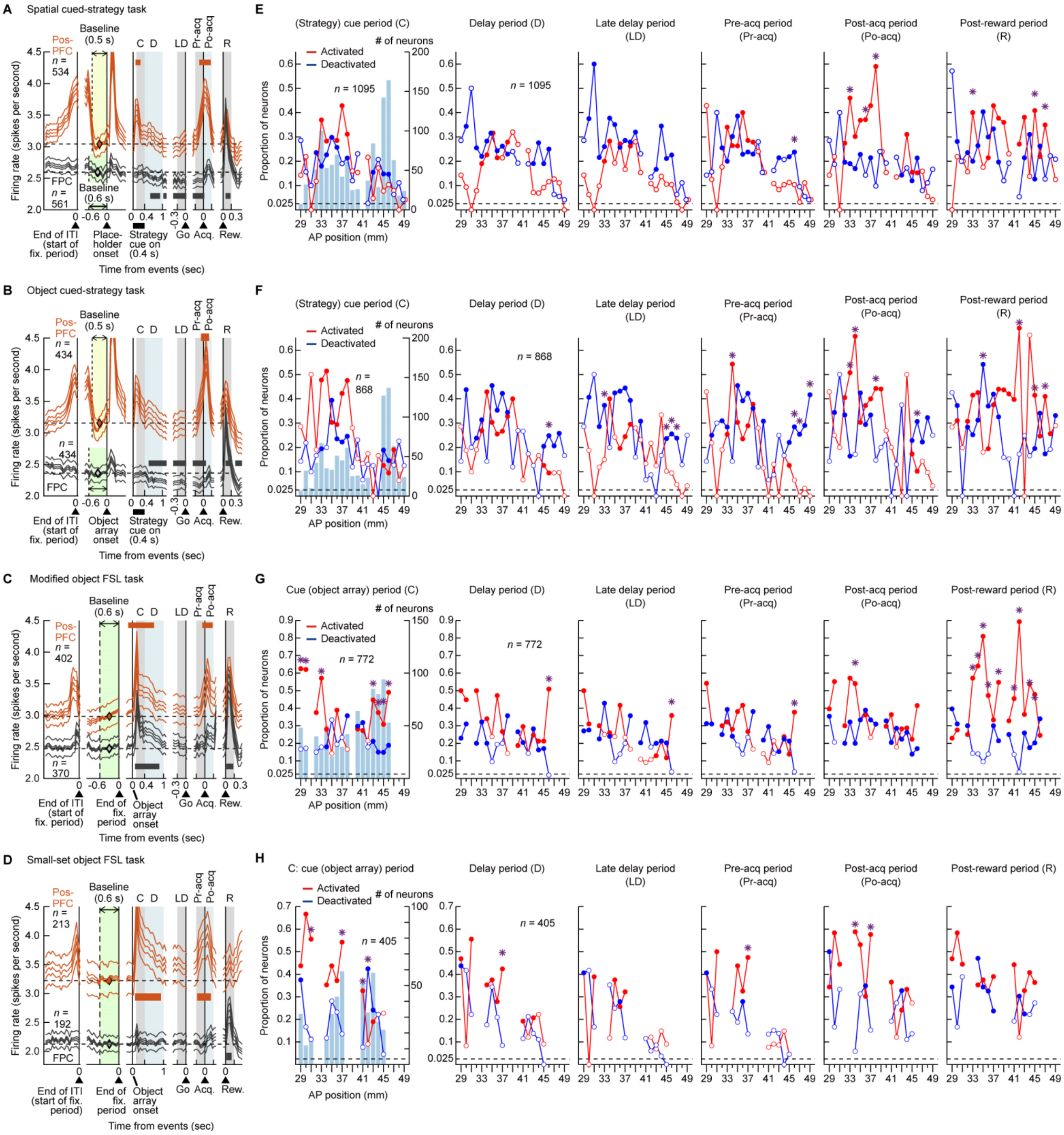

Figure S12 Task-specific breakdown of task-related activation/deactivation in the cued-strategy and few-shot learning tasks (Fig. 9C,D). (**A**) Spatial cued-strategy (CS) task. Population-averaged overall firing rate throughout the trial in the pos-PFC (orange) and FPC (gray). Conventions as in **Fig. 9C**. (**B**) Same as in **A**, but for the object CS task. (**C**) Same as in **A**, but for the modified few-shot learning (FSL) task (Experiment 3). Conventions as in **Fig. 9D**. (**D**) Same as in **C**, but for the small-set FSL task (Experiment 4). (**E**) Spatial cued-strategy (CS) task. Changes in the proportion of significantly activated (red) and deactivated (blue) neurons relative to baseline across AP locations, shown separately for each of the six task periods: (strategy) cue, delay, late delay, pre-acq, post-acq and post-reward. Conventions as in **Fig. 9G**. (**F** to **H**) Same as in **E**, but for the object CS (**F**), modified FSL (**G**), and small-set FSL (**H**) tasks.

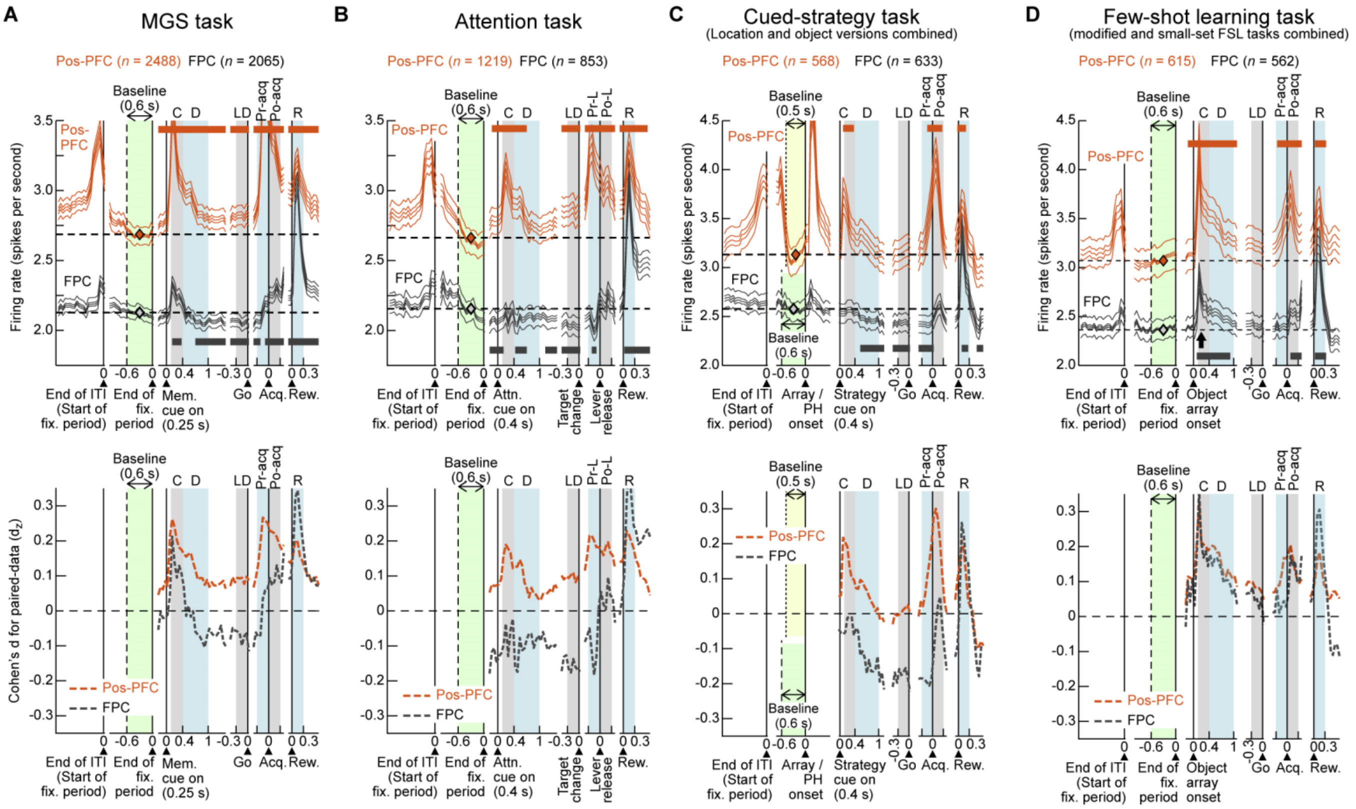

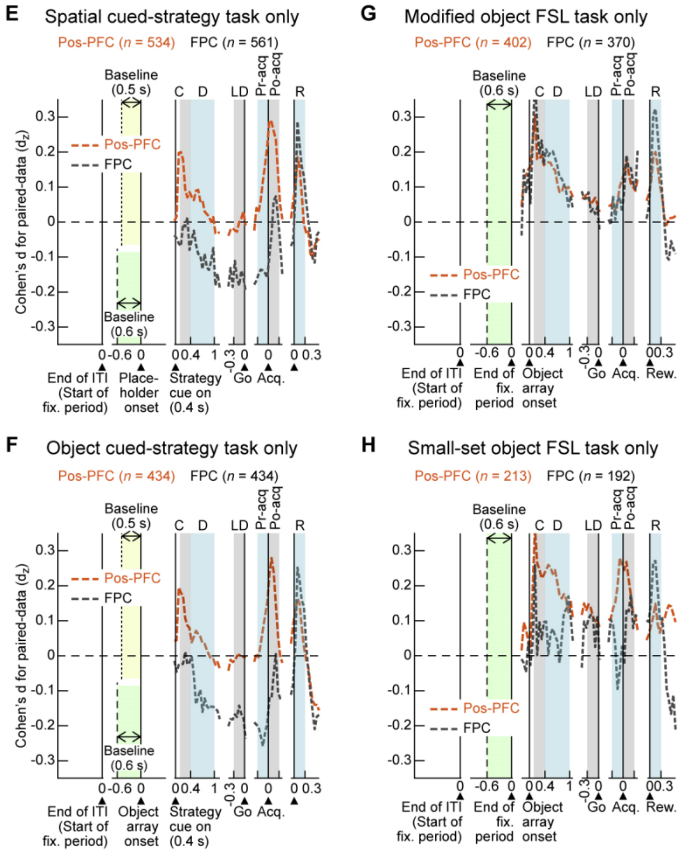

Figure S13 Time-resolved paired-samples Cohen's d_z measuring the effect size of task-related activation and deactivation in each task.
(**A**) MGS task. Top: population-averaged overall firing rate across the trial (**Fig. 9A** reproduced here for reference). Bottom: Cohen's d_z for pos-PFC (orange) and FPC (gray), aligned as in the top panel. Note that we did not take the absolute value of d_z in order to preserve the direction of the effect (negative d_z =task-related deactivation). For each bin, paired-samples Cohen's d_z was calculated by first computing, for each neuron, the paired difference (D) in firing rate between that bin and baseline, and then dividing the mean of these differences by their standard deviation (d_z = mean(D) / SD(D)). (**B–D**) Same as in (**A**), but for the attention task (**B**), cued-strategy task (**C**), and few-shot learning (FSL) task (**D**). (**E and F**) Breakdown of the cued-strategy results in (**C**) by individual tasks: spatial cued-strategy (**E**) and object cued-strategy (**F**) tasks. (**G and H**) Breakdown of the FSL results in (**D**) by individual tasks: modified FSL (Experiment 3) (**G**) and small-set FSL (Experiment 4) tasks (**H**).

Table S1

Number of neurons recorded from FPC and pos-PFC by experiment and monkey.

FPC neuron	Monkey K	Monkey T	Monkey H	Monkey Um	Total
Exp. 1	425	428			853
Exp. 2	115	101			216
Exp. 3	48		210	112	370
Exp. 4	52		140		192
Exp. 5	240			194	434
Exp. 5 Suppl. [a)]				199	199
FPC neuron total					2264

Pos-PFC neuron	Monkey K	Monkey T	Monkey H	Monkey Um	Total
Exp. 1	736	483			1219
Exp. 2	220				220
Exp. 3	145		257		402
Exp. 4	70		143		213
Exp. 5	151			283	434
Exp. 5 Suppl. [a)]				134	134
Pos-PFC neuron total					2622

Exp., experiment.

[a)] "Exp. 5 Suppl." refers to the additional 23 sessions in which neural activity during the spatial CS and MGS tasks was compared between the standard and delayed reward conditions (see **fig. S10**).
